# Supplementary material for: Compressed sensing for electron cryotomography and high-resolution subtomogram averaging of biological specimens
Source: Structure. 2022 Mar 3;30(3):408–417.e4. doi: 10.1016/j.str.2021.12.010 (PMC8919266; doi:10.1016/j.str.2021.12.010)
Supplement: Document S1. Figures S1–S4 [file mmc1.pdf]

Structure, Volume *30*

Supplemental Information

Compressed sensing for electron cryotomography and high-resolution subtomogram averaging of biological specimens

Jan Böhning, Tanmay A.M. Bharat, and Sean M. Collins

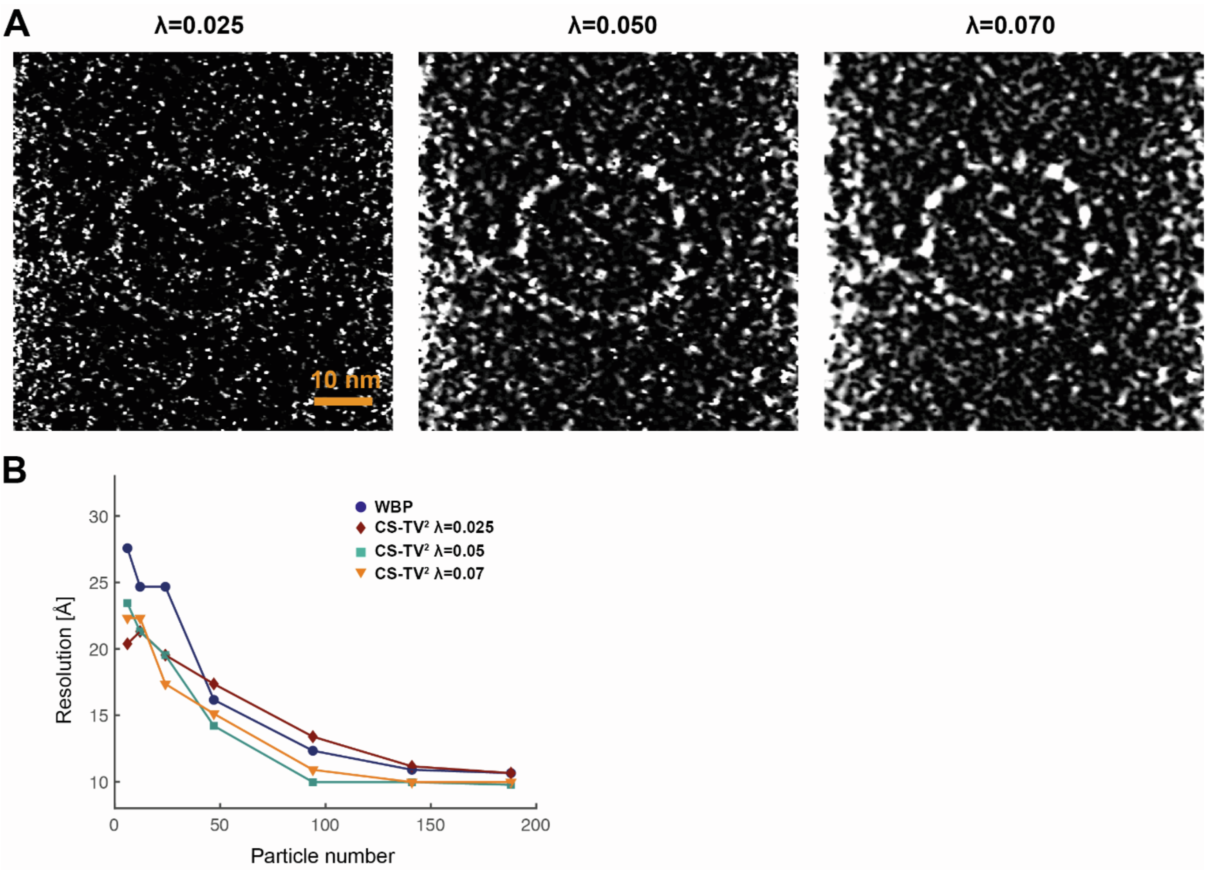

Figure S1. Testing regularization (λ) parameters for CS-TV^2 reconstruction, related to Figure 2.

A) Tomographic slices of reconstructed CS-TV^2 subtomograms with three different regularization parameters. λ=0.05 was selected for the majority of this study. B) Particle number-vs-resolution plot according to the gold-standard criterion indicating self-consistency between half-maps, with datasets reconstructed with different λ parameters. λ=0.05 was chosen as it showed the highest self-consistency between half maps for the full dataset. For resolutions higher than 11 Å, α-helical features could be clearly identified in the corresponding EM density. We speculate that different λ parameters may be suitable for different data amounts, i.e. more regularization might be useful for smaller data amounts and vice versa. While gold-standard FSC was used to find the ideal λ value, model-vs-map FSC were used to validate the results shown in Figure 2.

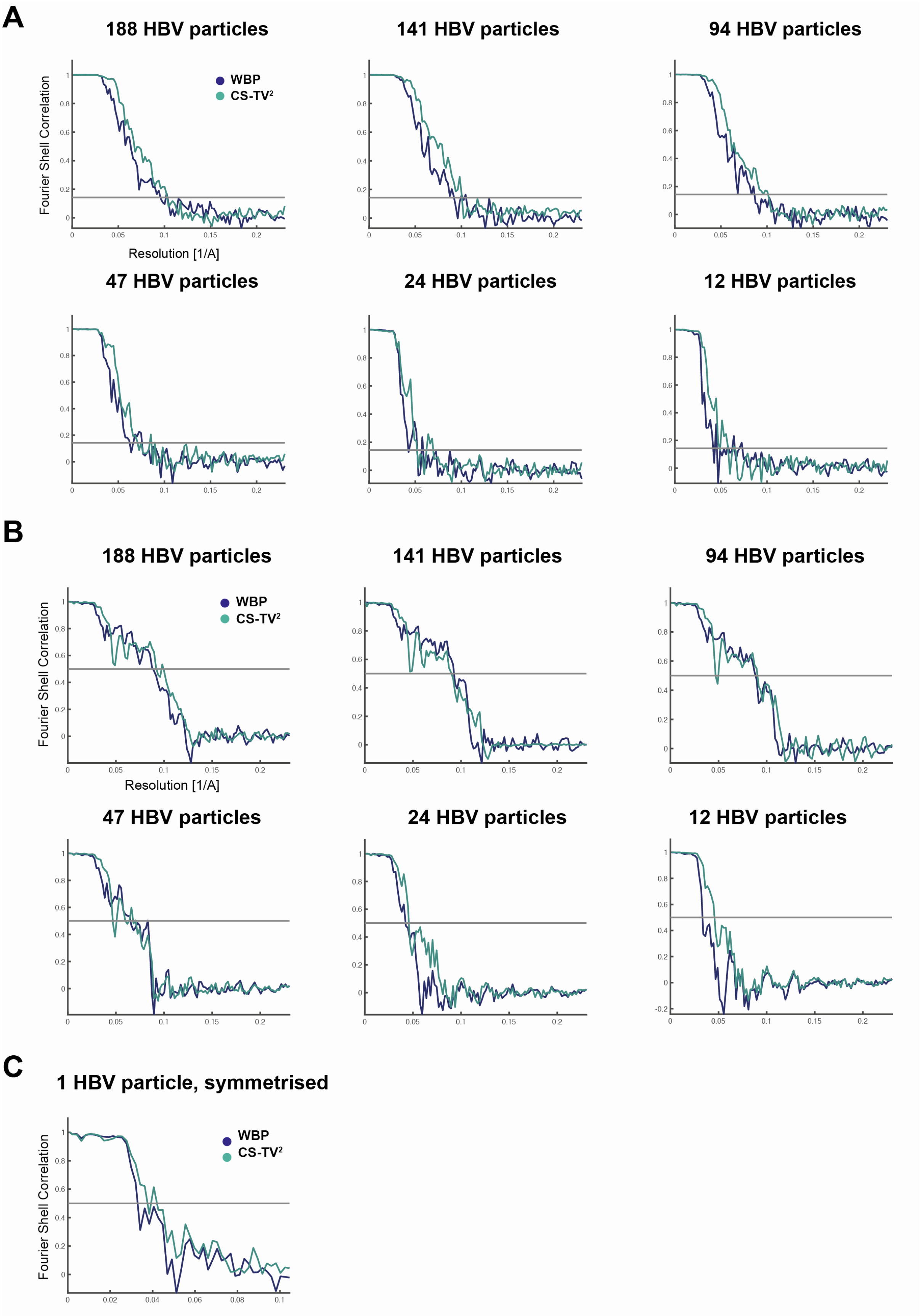

Figure S2. Gold-standard and model-vs-map FSC plots, related to Figure 3.

A) Gold-standard FSC plots for STA maps obtained from averaging of varying numbers of HBV capsid particles. The resolution criterion (FSC=0.143) is indicated. B) Model-vs-map FSC plots versus a density map created from an atomic model (PDB 6HTX). Horizontal line at 0.5 FSC is drawn. B) Model-vs-map FSC plot as in A) for a single subtomogram reconstructed with either WBP or CS-TV[2].

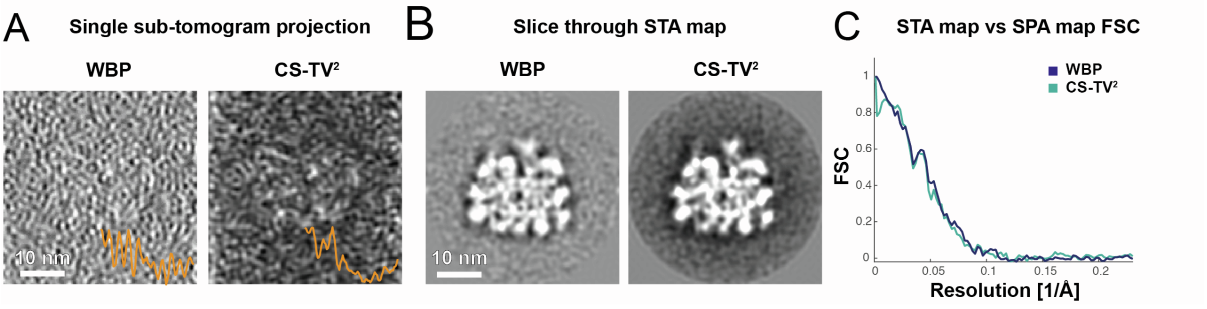

Figure S3. STA of an *S. cerevisiae* ribosome dataset (EMPIAR 10045) reconstructed with CS-TV^2 and WBP (control), related to Figure 3.

A) Projection image of a single ribosome subtomogram. CS-TV^2 allows clearer visualisation of the ribosome against the background. Radial averaging (orange line) shows significant variation in intensity for the WBP reconstructions, whereas increased intensity near the centre of the particle is visible for the CS-TV^2 reconstruction. B) STA map from all particles in the dataset, excluding those too close to the edge for CS-TV^2 reconstructions. C) FSC against a 3.7 Å cryo-EM map obtained through single particle analysis of the same preparation of specimen, indicating comparable performance between WBP and CS-TV^2. The same λ parameter (λ=0.05) as the HBV dataset was used for this dataset.

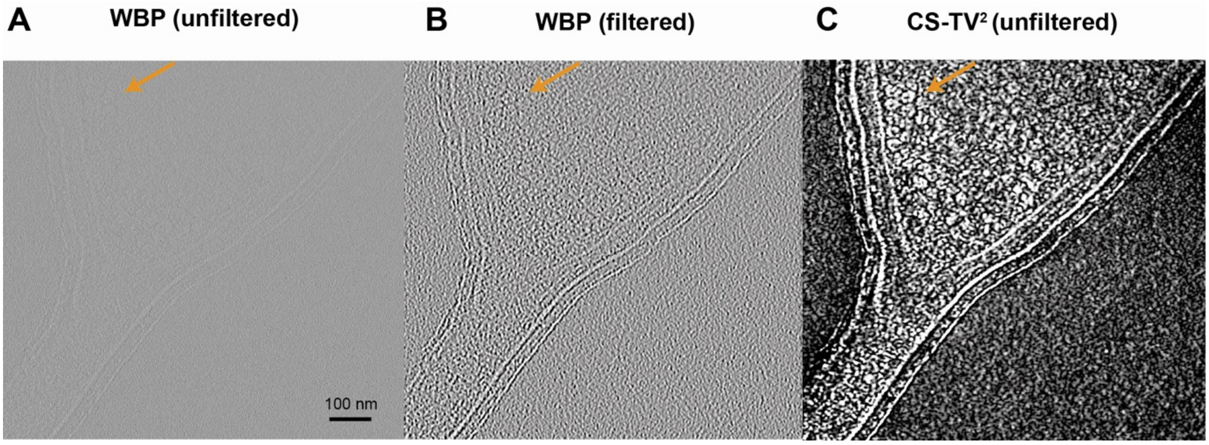

Figure S4. Cryo-ET of a *C. crescentus* cell, related to Figure 4.

A) Unfiltered WBP reconstruction, B) 3-sigma Gaussian and 45-Å lowpass-filtered WBP reconstruction, and C) unfiltered CS-TV^2-reconstruction (λ=0.0015) of the cell body of a *C. crecentus* cell. A putative polysome is indicated by an arrow. CS-TV^2 allows clear visualisation of individual ribosomes without any additional filter or loss of high-frequency information (shown in Figures 2-3).
