# Supplementary material for: Compressed sensing for electron cryotomography and high-resolution subtomogram averaging of biological specimens
Source: Structure. 2022 Mar 3;30(3):408–417.e4. doi: 10.1016/j.str.2021.12.010 (PMC8919266; doi:10.1016/j.str.2021.12.010)
Supplement: Document S2. Article plus supplemental information [file mmc5.pdf]

Structure

Resource

Compressed sensing for electron cryotomography and high-resolution subtomogram averaging of biological specimens

Graphical abstract

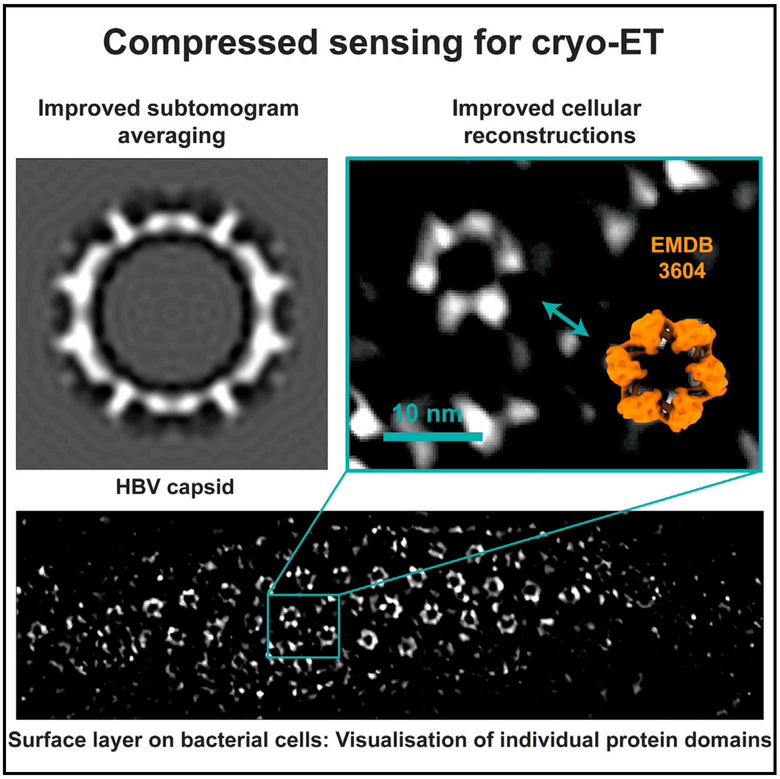

Authors

Jan Böhning, Tanmay A.M. Bharat, Sean M. Collins

Correspondence

tanmay.bharat@path.ox.ac.uk (T.A.M.B.), s.m.collins@leeds.ac.uk (S.M.C.)

In brief

Böhning et al. apply a compressed sensing (CS-TV^2) reconstruction algorithm utilizing 3D second-order total variation to cryo-ET data. The approach is benchmarked on purified and cellular biological specimens, showing that CS-TV^2 increases contrast within tomograms while retaining high-resolution information up to the secondary structure level.

Highlights

- Compressed sensing (CS-TV^2) for cryo-ET using 3D second-order total variation
- CS-TV^2 increases signal contrast while retaining high-resolution information
- Improved subtomogram averaging from CS-TV^2 reconstructions of small datasets
- Increased contrast and detail in CS-TV^2 reconstructions of cellular specimens

Böhning et al., 2022, Structure *30*, 408–417
March 3, 2022

© 2021 The Author(s). Published by Elsevier Ltd.

https://doi.org/10.1016/j.str.2021.12.010

Structure

Resource

Compressed sensing for electron cryotomography and high-resolution subtomogram averaging of biological specimens

Jan Böhning,[1] Tanmay A.M. Bharat,[1,2,4,*] and Sean M. Collins[3,*]
[1]Sir William Dunn School of Pathology, University of Oxford, Oxford OX1 3RE, UK
[2]Structural Studies Division, MRC Laboratory of Molecular Biology, Francis Crick Avenue, Cambridge CB2 0QH, UK
[3]School of Chemical and Process Engineering & School of Chemistry, University of Leeds, Leeds LS2 9JT, UK
[4]Lead contact
*Correspondence: tanmay.bharat@path.ox.ac.uk (T.A.M.B.), s.m.collins@leeds.ac.uk (S.M.C.)
https://doi.org/10.1016/j.str.2021.12.010

SUMMARY

Cryoelectron tomography (cryo-ET) and subtomogram averaging (STA) allow direct visualization and structural studies of biological macromolecules in their native cellular environment, *in situ*. Often, low signal-to-noise ratios in tomograms, low particle abundance within the cell, and low throughput in typical cryo-ET workflows severely limit the obtainable structural information. To help mitigate these limitations, here we apply a compressed sensing approach using 3D second-order total variation (CS-TV^2) to tomographic reconstruction. We show that CS-TV^2 increases the signal-to-noise ratio in tomograms, enhancing direct visualization of macromolecules, while preserving high-resolution information up to the secondary structure level. We show that, particularly with small datasets, CS-TV^2 allows improvement of the resolution of STA maps. We further demonstrate that the CS-TV^2 algorithm is applicable to cellular specimens, leading to increased visibility of molecular detail within tomograms. This work highlights the potential of compressed sensing-based reconstruction algorithms for cryo-ET and *in situ* structural biology.

INTRODUCTION

Cryoelectron tomography (cryo-ET) is an increasingly popular method for direct visualization of macromolecules in their native environment, which, together with subtomogram averaging (STA), allows structure determination of biological macromolecules (Beck and Baumeister, 2016; Briggs, 2013; Lučić et al., 2005; Wan and Briggs, 2016). Cryo-ET is often applied to specimens with a complex 3D arrangement, where standard 2D cryoelectron microscopy (cryo-EM) is not sufficient. Similarly, STA as a structure determination technique is often applied to specimens in cases where cryo-EM single-particle analysis is not feasible; for example, because of the pleomorphic nature of the sample (Briggs, 2013; Lučić et al., 2005). Uniquely, cryo-ET and STA can also be employed to visualize macromolecules and solve structures *in situ* within cells (Beck and Baumeister, 2016; Galaz-Montoya and Ludtke, 2017; Medeiros et al., 2018). Applications of cryo-EM and cryo-ET have increased steadily over recent years with advances in cryo-EM hardware and detector technology (Li et al., 2013; McMullan et al., 2009) in conjunction with increasingly powerful software for image processing (Bharat et al., 2015; Chen et al., 2019; Galaz-Montoya et al., 2015; Himes and Zhang, 2018; Kremer et al., 1996; Punjani and Fleet, 2021; Punjani et al., 2017; Tang et al., 2007; Tegunov and Cramer, 2019; Tegunov et al., 2021; Turoňová et al., 2017; Zhong et al., 2021).

Visualization of macromolecules in cells is extremely valuable because macromolecular interactions with the cellular environment are revealed, providing a wealth of important biological data (Melia and Bharat, 2018; Pfeffer and Mahamid, 2018; Villa et al., 2013). If the native structure of the macromolecule needs to be studied, then STA can be used to obtain higher-resolution reconstructions from cryo-ET data (Grünewald et al., 2003). STA is enabled by the fact that high-resolution information is preserved within the tilt-series images that are used to reconstruct tomograms (Lučić et al., 2013). The high-resolution information can be recovered by computational alignment and averaging of numerous tomographic subvolumes containing identical copies of the target macromolecule, called subtomograms (Beck and Baumeister, 2016; Briggs, 2013). The potential of STA has been impressively demonstrated in recent years, with reports of maps from which the atomic structures could be directly interpreted (Himes and Zhang, 2018; Schur et al., 2016; Tegunov et al., 2021). Despite these recent successes, there is large potential for improvements and future applications. Most recent applications of cryo-ET and STA resulting in near-atomic resolution focused on intrinsically thin specimens with a large, abundant macromolecular assembly used for STA. Apart from a handful of such success stories, typical cellular cryo-ET resolutions are between 10 and 50 Å, where integrating orthogonal information about the macromolecule of interest is often

 © 2021 The Author(s). Published by Elsevier Ltd.
This is an open access article under the CC BY license (http://creativecommons.org/licenses/by/4.0/).

CellPress
OPEN ACCESS

required to reach a satisfactory biological conclusion (Allegretti et al., 2020; Ghosal et al., 2019; Hoffmann et al., 2019; Shi et al., 2019; Watanabe et al., 2020; Weiss et al., 2019).

The quality of the cryo-ET data ultimately governs the success of any study because it strongly influences, first, the possibility of target detection in crowded cellular tomograms and, second, the quality of the extracted subtomograms, thus determining the resolution of the final structure after STA refinement. Tomographic reconstructions from 2D tilt-series projection images are performed after deducing the geometrical relationship between tilt-series images. This is often enabled by assessing positions of gold fiducial markers in the images, along with prior knowledge of specimen tilt applied during data collection. By knowing these parameters, tomographic reconstruction can then be performed using various algorithms (Sorzano et al., 2017). If the study requires higher-resolution structural analysis (i.e., when STA is needed), typically large amounts of data are used to overcome the low signal-to-noise ratio (SNR). This procedure is well established but often requires thousands of subtomograms and hundreds of tomograms, which can create a huge bottleneck in STA, depending on the abundance of the target macromolecular complex (Böhning and Bharat, 2021). First, sample preparation for *in situ* structural biology often involves focused ion beam (FIB) milling, during which material is ablated with an ion beam above and below the region of interest to create thin cellular slabs that are amenable for cryo-ET (Marko et al., 2007; Villa et al., 2013). Even with recent advances in automation of the FIB milling process (Klumpe et al., 2021; Zachs et al., 2020), this process is time-consuming and limited in throughput. Secondly, cryo-ET data acquisition is much slower than in single-particle cryo-EM. For *in situ* structural biology applications, therefore, it is of utmost importance to develop methods to improve solving structures of macromolecules with limited datasets at hand to reach the long-standing goal of "visualizing the sociology" of the cellular proteome (Baumeister, 2002).

To this end, there is an array of reconstruction methods available to produce high-quality tomograms (Sorzano et al., 2017). Commonly used algorithms include the simultaneous iterative reconstruction technique (SIRT) (Agulleiro and Fernandez, 2011; Gilbert, 1972; Lučić et al., 2005), the algebraic reconstruction technique (ART) (Gordon et al., 1970), and algorithms derived thereof, such as discrete ART (Batenburg and Sijbers, 2007) and simultaneous ART (SART) (Andersen and Kak, 1984). Such algorithms iteratively reduce the differences between calculated projections of the tomogram and the tilt series, which often results in increased sample contrast. The amplification of low-resolution features, which increases visibility, coincides with a loss of high-resolution features below the noise levels of tomograms (Wan and Briggs, 2016). Some algorithms have been developed in an effort to retain high-resolution features while enhancing low-resolution contrast, such as the iterative nonuniform fast Fourier transform reconstruction (INFR) (Chen and Förster, 2014), super-sampling SART (Kunz and Frangakis, 2014), and the progressive stochastic reconstruction technique (PSRT) (Turoňová et al., 2015). These algorithms showed an impressive boost in STA resolution to 20–30 Å. More recently, model-based iterative reconstruction (MBIR) has been employed for cryo-ET, where a prior model of the unknown 3D object is employed to guide reconstruction. The algorithm has been shown to produce higher-contrast reconstructions (Yan et al., 2019), and benefit at ~15-Å resolution has been suggested in STA, although the algorithm requires strong high-pass filtering of the resulting reconstructions for STA because of the prevalence of low spatial frequencies within reconstructed tomograms.

In materials science applications, atomic-resolution tomography has been achieved at substantially higher doses and SNRs using equally sloped tomography methods (Scott et al., 2012) as well as mixed real and Fourier space iterative methods (Pryor et al., 2017; Yang et al., 2017). This type of dual-space algorithm, augmented by use of constraints such as non-negativity and total variation (TV) regularization, has recently also seen application in cryo-ET, with improvements in contrast and noise level (Geng et al., 2021). Machine learning approaches include dictionary learning, and neural network approaches have also shown promise for enhancing reconstruction quality in the physical sciences (AlAfeef et al., 2016; Bladt et al., 2015; Liu et al., 2014). For biological applications, the electron dose that can be applied is severely limited because of the radiation sensitivity of specimens. For such samples, ideally, tomographic reconstruction algorithms would increase low-resolution contrast while preserving high-resolution frequencies up to the secondary structure level and beyond.

A promising method that has been shown to reduce data requirements in electron tomography is compressed sensing (CS) (Leary et al., 2013; Saghi et al., 2011). This image processing technique allows high-fidelity reconstructions of signals in cases where sampling is limited, which is uniquely applicable to the data-limited case of cryo-ET. This approach has seen widespread adoption in materials science at resolutions greater than 1 Å, with particular emphasis on the reduction in the number of projection images required for reconstructions of isolated structures (Leary et al., 2013). Applications of CS in electron tomography of biological samples, where SNRs are typically much lower than in materials science, have been far fewer (Deng et al., 2016; Geng et al., 2021; Guay et al., 2016; Li et al., 2020; Saghi et al., 2016), building on work exploring cryo-ET with regularization tailored to reducing missing wedge artifacts (Aganj et al., 2007) as well as CS-based image inpainting of high-contrast objects, such as fiducial markers (Song et al., 2012). Although CS-based STA has been reported at a resolution of several nanometers for ribosome samples (Deng et al., 2016), its ability to retain fine molecular detail at the level of secondary structure elements of macromolecules in cryo-EM applications and its use for STA of limited datasets and for direct visualization of macromolecules in the cellular environment remain undetermined.

In this study, we applied a CS approach to cryo-ET reconstructions of biological specimens, leveraging CS algorithms using 3D second-order TV (TV^2) (Collins et al., 2019). This work is motivated by significant recent advances in applying higher-order TV methods in electron tomography (Huber et al., 2019; Jacob et al., 2021; Sanders et al., 2017), with the aim of establishing viability of higher-order TV methods for cryo-ET and STA. In comparison with earlier CS-ET approaches in cryo-ET (Deng et al., 2016), our CS-TV^2 approach is compatible with dense images of high-resolution structures, uses a real space projection operator avoiding interpolation requirements in Fourier space algorithms (Goris et al., 2012), and accounts for the full three-dimensionality of the object for improved reconstructions

(Haberfehlner et al., 2014) by exploiting the connectivity inherent in biological structures. We tested the CS-TV^2 algorithm for tomographic reconstruction and found that it improves the SNR in tomograms of a wide range of specimens, facilitating visualization of macromolecules in raw tomograms. Furthermore, CS-TV^2 tomograms preserve secondary structure information and support high-resolution STA of biological macromolecules. We provide a detailed comparison of CS-TV^2 with weighted back-projection (WBP) using STA of purified specimens. We find that CS-TV^2 outperforms WBP at small subtomogram numbers (small datasets) while providing comparable results for medium-sized datasets where secondary structure elements could be resolved. We further applied our algorithm to cellular specimens to highlight the ability of CS-TV^2 tomographic reconstructions to remove noise and increase the visibility of macromolecular features at the level of small protein domains. We propose that further development in CS algorithms will be beneficial for cryo-ET, with gains in direct visualization of macromolecules in tomograms as well as in STA of small datasets, which are typical for *in situ* structural studies.

RESULTS

Mathematical basis for the approach

The key principles of CS and TV regularization have been reported in several contexts, with detailed studies of applications to electron tomography (Leary et al., 2013), cryo-ET (Deng et al., 2016), as well as higher-order TV for electron tomography (Sanders et al., 2017). The central tenets are reviewed briefly here, with a specific elaboration of the TV^2 approach applied to cryo-ET data. The mathematically rigorous foundations of CS are considered in the context of undersampled data. Undersampled data refers to a limited set of measurements that would be insufficient to recover a tomographic reconstruction with high fidelity while maintaining strict generality. Such a general sampling criterion assumes that the object may have intensities distributed in any possible 3D arrangement. CS instead uses the fact that objects of interest are highly structured and can therefore be represented with high fidelity with only a few coefficients, which is also the principle of image compression. In contrast to compression of a dense image, CS seeks to match a limited number of measurements to identify these coefficients directly and therefore requires far less data to complete a reconstruction.

The CS approach depends on the sparsity of the object in this compressed representation where few coefficients are needed. CS further depends on the incoherence between the structure of sampling or distribution of the measurements (i.e., the tilt-series data) and the mathematical description of the measurement process (i.e., the Radon transform) in cryo-ET. In many CS applications, this incoherence is established by randomization in the measurements taken. In electron tomography, these requirements appear to follow the asymptotic incoherence properties for effective sampling in CS (Adcock et al., 2017), given the distribution of measurements as slices through Fourier space, and the CS framework has demonstrated success in numerous ET applications.

The CS-ET algorithm can be cast as a regularized reconstruction problem of the form

$$f(\boldsymbol{r}) = \arg\min_{\boldsymbol{r}} \left\{ ||\widehat{P}f(\boldsymbol{r}) - \Gamma^{exp}||_{\ell_2}^2 + \lambda ||\psi\{f(\boldsymbol{r})\}||_{\ell_1} \right\},$$

where $f(\boldsymbol{r})$ is the reconstruction volume over 3D coordinates $\boldsymbol{r}$, $\widehat{P}$ is the projection operator, Γ^{exp} is the experimental data, λ is a constant weighting factor, and $\psi\{f(\boldsymbol{r})\}$ is a transform applied to the volume to a selected domain requiring few non-zero coefficients to represent the object (i.e., the sparse domain). The notation $||\cdot||_{\ell_p}$ refers to the ℓ_p-norm for the data. The first term accounts for a least-squares data fidelity (the definition of the ℓ_2-norm), and the second term incorporates the sparsity constraint to recover the solution or reconstruction with the intended structure (sparsity) that optimally accounts for the experimental data. The weighting factor λ is set relative to the data fidelity term and so will vary with inherent sparsity of the object in the transform domain, the size of the tilt-series images, the projection image intensities, and the noise level in the data, along with associated errors and any inconsistencies in the re-projected reconstruction and the tilt-series data. In contrast to pre- or post-reconstruction image processing, CS reconstructions can be thought of as the best reconstructions for a given weight assigned to denoising (incorporating the true sparsity and the noise in the data). The ℓ_1-norm, the magnitude rather than square of coefficients in the transform domain, promotes sparsity while enabling practical algorithmic implementation. In CS-TV^2 reconstructions, the sparsity term is replaced by $\lambda\, TV^2\{f(\boldsymbol{r})\}$. TV operations, although not identical to the ℓ_1-norm, have similar properties and can be described as capturing the magnitude of the estimates of the finite image gradients. The second-order gradients imply an object that is piece-wise linear in structure. This choice of transform domain is particularly appropriate for high-resolution structures that are dense (i.e., they contain many non-zero intensities) images with varying intensities (i.e., not piece-wise constant, as expected for TV regularization). Crucially, the intrinsic sparsity of a 3D object and the associated relative weighting of the transform term apply in 3D for the calculated CS-TV^2 term in our implementation.

Figure 1 presents a schematic overview of this approach applied to a 2D phantom dataset, derived from a model volume of a hepatitis B (HBV) triangulation number (T) = 4 capsid with noise added. Figure 1A highlights a conventional approach, with projection of the phantom to generate a noisy sinogram and reconstruction by WBP. Figure 1B illustrates the modifications for CS-TV^2. The phantom is assumed to be well approximated by a piece-wise linear representation, meaning that only a small number of spatial second-order gradients are non-zero. Cryo-ET samples for STA are unlikely to exhibit first-order TV characteristics (piece-wise constant) because of fine-scale changes in density and intensity, distinct from TV-sparse features exhibiting homogeneous density with sharp boundaries. The second-order gradients of the phantom show a restricted number of non-zero intensities. The distribution is similar to the image itself; however, the identity transform (i.e., taking the sparsity of the image itself) as an alternative does not promote reconstructions with 3D connectivity in the same way as 3D CS-TV^2. In conjunction with the data fidelity term, which seeks solutions that match the sinogram, the CS algorithm iteratively calculates the TV^2 representation and balances these contributions in the overall minimization problem.

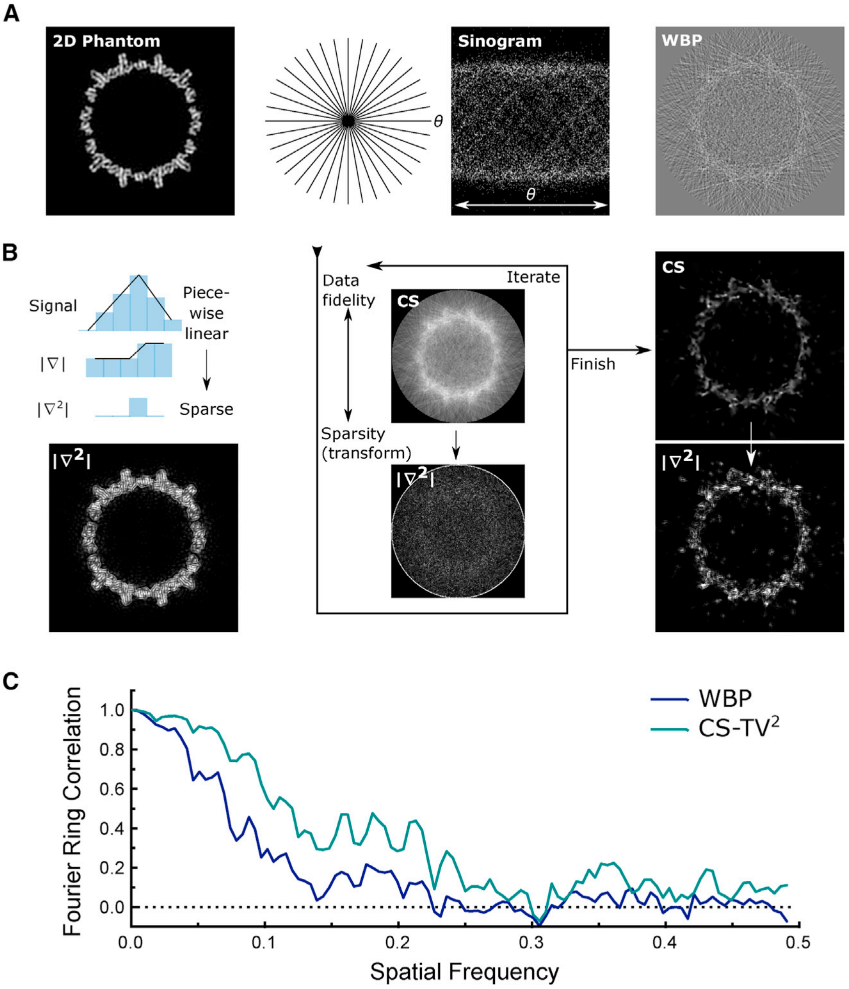

Figure 1. Illustration of the key steps in compressed sensing using 3D second-order total variation (CS-TV^2) ET

(A) A 2D phantom (ground truth) is projected to give a tilt-series dataset. The projection data at each sample orientation form a sinogram used to calculate a WBP reconstruction.

(B) CS involves identifying a sparse representation, depicted in terms of the non-zero elements (blue) in the second-order gradient magnitude for the ground truth; TV^2 promotes sparsity in the second-order gradient (∇^2) of the image. An iterative algorithm balances data fidelity and sparsity in the transform domain, yielding a final reconstruction with optimized sparsity and fidelity to the projection data.

(C) Fourier ring correlation (FRC) curves for WBP and CS-TV^2 reconstructions of the phantom. The horizontal dashed line marks zero on the vertical axis.

In the presence of high noise levels, the CS algorithm recovers a phantom reconstruction with a flat near-zero background and high visibility of the structural features. Figure 1C further illustrates the improvement in reconstruction quality by plotting Fourier ring correlation (FRC) curves referenced to the ground truth phantom, showing a substantial extension of the FRC profile in spatial frequencies recovered by the CS-TV^2 algorithm.

Applying CS-TV^2 tomographic reconstruction to biological cryo-ET data

To test the mathematical approach described above, and to assess the level of structural details that can be resolved in biological samples by CS-TV^2 reconstructions, we used a cryo-ET dataset of HBV T = 4 capsid particles, which has been shown previously to yield subnanometer-resolution STA structures (Bharat et al., 2015). We used the CS-TV^2 algorithm for tomographic reconstruction of subtomograms from the tilt-series data, which was contrast transfer function (CTF) compensated by phase flipping. Data from three distinct tomograms, containing 188 particles of HBV capsids, were used for tomographic reconstruction.

We performed CS-TV^2 reconstruction of the dataset using different regularization parameters (λ). For the majority of this study, a dataset reconstructed with a parameter of λ = 0.050 was used, which showed the highest level of self-consistency between half-maps (gold-standard Fourier shell correlation [FSC]) for the full dataset of 188 HBV capsid particles (Figure S1). The reconstructed CS-TV^2 subtomograms show considerably increased contrast compared with the WBP control, and the molecular envelope of the HBV capsid is clearly recognizable in the reconstruction (Figure 2A; Video S1). The level of noise in the WBP tomogram is significantly higher, making straightforward identification of the molecular envelope in the data difficult by a visual inspection of the reconstructed volume. This is underlined by radial averaging of the intensities, where the CS-TV^2 reconstruction shows a clear peak corresponding to the capsid density compared with WBP, where this peak is comparable with the background gray values, indicating a lower SNR (Figure 2A).

As a further test of the data quality, we centered a reconstructed subtomogram of an HBV particle and applied its internal icosahedral symmetry (I2 in RELION) (Scheres, 2012). In the symmetrized volume, the envelope of the HBV capsid became clearly recognizable for the CS-TV^2 reconstruction, with markedly lower levels of noise visible (Figure 2B). This effect is also observed in isosurfaces of the symmetrized volume displayed at the same contour level (Figure 2C), showing a clear molecular envelope of the particle in the CS-TV^2 reconstruction.

STA with CS-TV^2-reconstructed tomograms

STA maps from the CS-TV^2 dataset showed clear α-helical densities after B-factor sharpening (Figures 3A and 3B; Video S2). CS-TV^2 appeared to outperform WBP according to gold-standard estimation for the full dataset (Figure S2A). To confirm this observation, we performed a comparison against a density generated from an atomic model (Böttcher and Nassal, 2018) in a model-versus-map FSC measurement, with the atomic model (PDB: 6HTX) representing the ground truth (Figure 3C). Using a resolution criterion of 0.5 cutoff, model-versus-map FSC estimated a

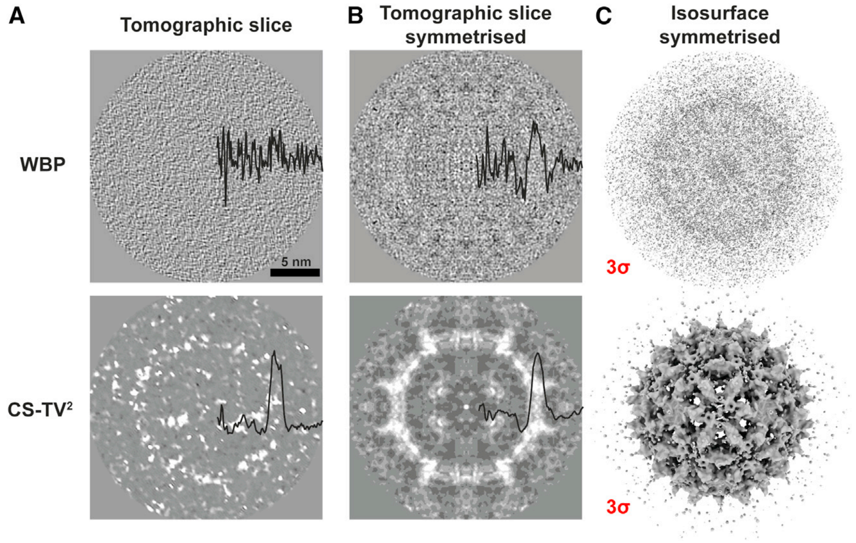

Figure 2. CS-TV^2 reconstruction of hepatis B virus (HBV) capsids

(A) A single HBV capsid particle reconstructed using WBP (top) and CS-TV^2 reconstruction (bottom). Radially averaged intensities of the reconstructions were normalized and plotted onto the image.

(B) The same HBV particle as shown in (A), with icosahedral (I2) symmetry applied. Radially averaged intensities were normalized and plotted onto the image.

(C) Symmetrized HBV particle as in (B), displayed as isosurfaces at 3σ contour level.

See also Figure S1.

resolution of 10.9 Å for CS-TV^2 and 11.4 Å for WBP. This experiment further indicates that secondary structure features are preserved within the CS-TV^2 reconstructions and can be resolved in STA maps.

Given the considerably higher SNR of CS-TV^2 subtomograms compared with WBP, we hypothesized that CS-TV^2 would be particularly advantageous for STA at reduced dataset sizes, where WBP averages may suffer from low SNR and poor refinement. Therefore, we next tested the effect of reduced dataset sizes on STA refinement. Using 50% of the dataset, the CS-TV^2 reconstructions allowed an 11.2-Å-resolution map to be resolved, compared with 11.4 Å for WBP, as estimated by model-versus-map FSCs (Figure S2B), indicating comparable performance. We then similarly tested STA refinements with reduced dataset sizes (Figure S2B). At ~6% of the dataset, the resolution, as estimated by model-versus-map FSC, was significantly improved for CS-TV^2 compared with the WBP control (Figure 3C): 20.8-Å resolution for the CS-TV^2 STA map compared with 31.2 Å for WBP. This indicates that employing CS-TV^2 reconstruction for small particle numbers (small dataset sizes) can improve the quality of STA maps, which is potentially important for applications such as integrative molecular modeling, a commonly used technique in *in situ* structural biology. Finally, the model-versus-map FSC of just a single centered and symmetrized HBV particle reconstructed with CS-TV^2 shows higher correlation with an atomic model compared with WBP (Figure S2C), indicating that the gain in contrast (Figure 2) correlates with increased resolution of molecular features. Because the aligned tilt series used for tomographic reconstruction is the same for CS-TV^2 and WBP, the high-resolution information of the target macromolecule in the source data is the same. We thus postulate that the improved correlation to the atomic model for STA maps made with CS-TV^2 data is due to elimination of noise within the reconstruction. This may also explain why CS-TV^2 reconstructions resemble the target macromolecule more than WBP reconstructions when performing STA with small particle numbers.

To demonstrate that the method is reproducible with other datasets, we reconstructed particles from a publicly available dataset (EMPIAR-10045) (Bharat and Scheres, 2016) containing purified 80S ribosomes from *S. cerevisiae*, using the same regularization parameter of λ = 0.050 used for reconstruction of HBV capsid particles (Figures 2 and 3). Although the CS-TV^2 reconstruction of single subtomograms remain noisy, CS-TV^2 allows improved visualization of the particle envelope in a projection image (Figure S3A, radial average in orange) compared with WBP. Performing STA on the dataset, we obtained a map for the CS-TV^2 reconstruction comparable with the WBP control, indicating no significant loss of features in the CS-TV^2 case. Because no atomic model of exactly the same sample is publicly available, we measured the FSC against a 3.7-Å single-particle cryo-EM density of the same specimen from the same source (Bharat and Scheres, 2016). This FSC measurement against the high-resolution map showed that CS-TV^2 reconstruction quality was comparable with WBP (Figure S3C), indicating that similar performance can be obtained without significant re-optimization of the λ parameter. This experiment agrees with our results on HBV particles shown in Figures 2 and 3.

The λ parameter is expected to be sensitive to the molecular structure (i.e., the true sparsity of the object, which is unknown experimentally) as well as the size and number of the tilt-series images, the SNR determined by the acquisition parameters and detector, and any residual inconsistencies in the tilt-series data (e.g., misalignments or deviations from the projection requirement). The results here show that the choice of λ is reasonably robust for very different molecular geometries when acquired under otherwise similar conditions, such as those generally used for cryo-ET.

Using CS-TV^2 tomographic reconstructions on cellular specimens with a complex 3D arrangement for improving visualization of biological detail *in situ*

Because CS-TV^2 reconstruction considerably enhanced the visibility of the molecular envelope in the raw data for purified specimens shown above, we decided to test this algorithm on a more complex 3D specimen. We processed tilt-series data from the stalk of a *Caulobacter crescentus* cell (Bharat et al., 2017; Sulkowski et al., 2019). The stalk is a cellular appendage, a continuation of the cytoplasm, which is encapsulated by a surface layer (S-layer), made up of pseudo-hexamers of a protein called RsaA (von Kugelgen et al., 2020). Because no STA was performed, and visualization of cellular macromolecular complexes was the goal, the λ parameter (λ = 0.005) was chosen according to visual

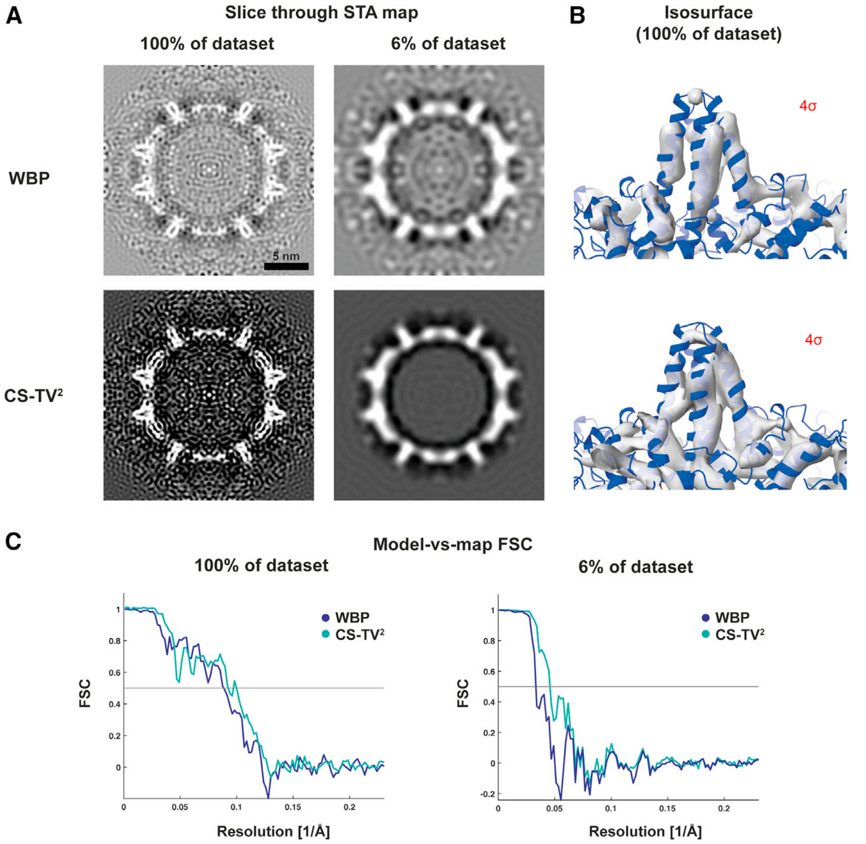

Figure 3. STA of CS-TV^2-reconstructed HBV capsids

(A) Subtomogram averages of the full dataset (188 capsid particles) and ~6% of the dataset (12 capsid particles) from WBP (top) and CS-TV^2-reconstructed (bottom) subtomograms, as visualized in IMOD.

(B) Isosurface representation of subtomogram averages shown in (A) at 4σ isosurface contour level, along with the atomic model (PDB: 6HTX) rigid body fitted into the density.

(C) Model-versus-map FSC plots against an atomic model of HBV representing the ground truth (PDB: 6HTX).

See also Figure S2.

inspection. The significantly different value of λ used here was attributed to the very different object structure, image size used in reconstructions, and experimental acquisition parameters relative to the STA datasets.

A WBP tomographic reconstruction shows low SNR and reduced contrast where the S-layer hexamers are not visible in unprocessed and unfiltered tomograms (Figure 4A). Using strong low-pass (~45 Å) and Gaussian filtration allows visualization of some RsaA N-terminal domain hexamers (Figure 4B, marked). The CS-TV^2 reconstruction (Figure 4C) not only makes individual RsaA hexamers clearly visible against the background without any image processing, such as filtration applied after reconstruction, but also resolves the molecular envelope of the ~25-kDa monomers of the RsaA N-terminal domain within individual hexameric densities (Figure 4C; Video S3). Comparison of the RsaA S-layer subnanometer-resolution STA map (EMD-3064; Figure 4D) against the unfiltered CS-TV^2 tomogram shows that the shape of the small domain is faithfully replicated in the tomographic density.

As a further test, we also performed CS-TV^2 reconstruction of tomographic data from the cell body of a *C. crescentus* cell. The reconstructed tomograms confirm that CS-TV^2 is ideally suited to elevate visibility of cellular features such as ribosomes, membranes, and the S-layer, which are extremely difficult to detect in the unfiltered WBP reconstruction. Strong low-pass (~45 Å) and Gaussian filtration of the WBP reconstruction recovers some of these features for visualization, but, by definition, the higher-resolution Fourier components are lost (Figure S4).

These results indicate that CS-TV^2 reconstructions are helpful at denoising cellular data and particularly useful for resolving the molecular envelopes of protein complexes within their cellular environment. The unique nature of CS-TV^2, which enhances low-frequency features but preserves high-frequency information, means that the tomograms can be used for visualization and for STA structure determination directly.

DISCUSSION

A common issue for methods that increase contrast in cryo-ET is that, by enhancing low-frequency information and eliminating noise, high-resolution information is lost (Wan and Briggs, 2016). In this study, we successfully applied a CS-TV^2 approach to biological cryo-ET data, showing that it not only leads to considerable increase in contrast of tomograms but also preserves high-resolution information up to the secondary structure level. Our results show that employing CS-TV^2 for tomographic reconstruction provides improved STA refinements from small datasets, leading to cryo-ET densities that accurately represent the ground truth. Improved STA maps from less data are important for *in situ* structural studies because these may enable improved integrative modeling (Rout and Sali, 2019), which is typically employed to interpret cellular cryo-ET data on a structural level. The higher-resolution densities produced by STA of CS-TV^2 data could help produce higher-fidelity molecular models, of significant value to cell biologists. Furthermore, requiring less data could alleviate bottlenecks of cellular cryo-ET, which suffers from notoriously low throughput. Although some throughput issues are currently being tackled in ground-breaking studies that produce previously unseen amounts of cellular data through automation of FIB milling procedures (Allegretti et al., 2020; Klumpe et al., 2021; Zachs et al., 2020), the amount of instrument time required for cryo-ET makes many projects hard to realize for many laboratories. The data amount required for STA should be reduced with the use of CS-TV^2, which will save valuable time on expensive cryo-electron microscopes (Figure S2). Thus, CS-TV^2 reconstruction will be another arrow in the quiver of the structural biologist to tackle these issues.

We also demonstrated in this study that CS-TV^2 can enhance the visibility of macromolecules in cellular cryo-ET data. Finding

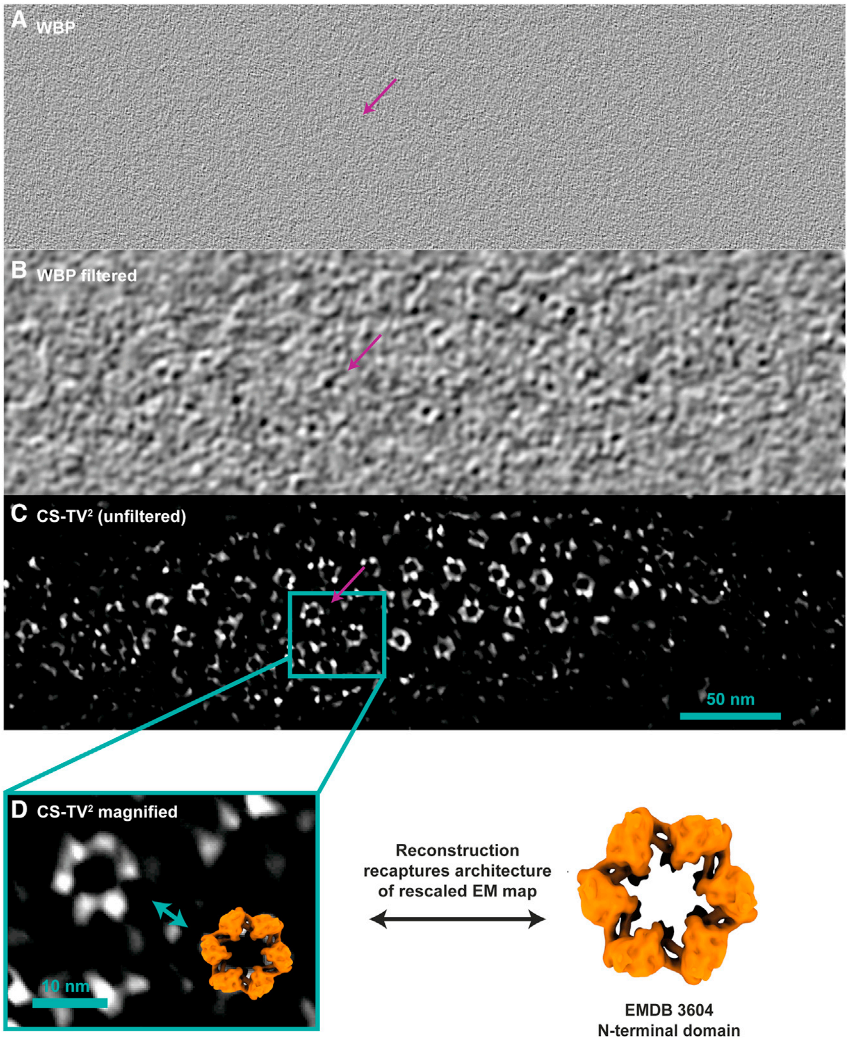

Figure 4. CS-TV^2 tomographic reconstruction improves visibility of macromolecules in tomograms

(A–C) The same tomographic slice of (A) an unfiltered WBP tomogram, (B) a low-pass-filtered (45 Å) and Gaussian-filtered WBP tomogram, and (C) an unfiltered CS-TV^2 reconstruction (λ = 0.005) of a *C. crescentus* stalk covered by an S-layer. Although a low SNR in the WBP reconstruction does not allow clear visualization of individual hexameric RsaA molecules making up the *C. crescentus* S-layer, the CS-TV^2 reconstruction allows clear visualization of hexamers and their individual monomers (~25 kDa).

(D) An appropriately rescaled isosurface of an EM map of the RsaA N-terminal domain (EMD-3604) was overlayed in the magnified view for comparison, showing faithful recovery of molecular detail.

See also Figure S4.

macromolecules in cells and tissues is notoriously difficult (Melia and Bharat, 2018; Wang et al., 2011) and often requires additional experiments, such as immunolabeling or cryo-fluorescence microscopy. Increased SNR in tomograms may reduce the reliance on these additional steps, allowing more straightforward identification of target macromolecules by visual inspection of the density or by utilizing a template matching approach (Frangakis et al., 2002; Wu et al., 2019). The revealed molecular detail, enabled through the increase in contrast while maintaining high-frequency resolution information, allows direct interpretation of the data without the need for additional filtration or denoising procedures.

A disadvantage of the method is that CS-based algorithms are more computationally expensive than other commonly used reconstruction techniques, especially because several reconstructions with different λ parameters may have to be probed for optimal results. In experimental CS methods, determination of the optimal value of λ is a persistent problem. The demonstration here that appropriate selection of λ can be performed by evaluation of the FSC could facilitate CS-ET methods beyond biological structures. The illustration of retained high-resolution information at a low SNR suggests that much lower SNRs may be used for electron tomography in physical sciences applications where reduced doses may be required for beam-sensitive materials with or without cryogenic workflows. In cryo-ET applications, a suitable value of λ can be determined from a small subset of reconstructions prior to parallel reconstructions of multiple or large volumes. The increasingly common use of graphical processing units (GPUs) in cryo-EM image processing will greatly accelerate reconstruction speed because the algorithm can be easily parallelized and ease the widespread use of CS algorithms. Improved integration into previous STA pipelines could also increase the usability of the CS-TV^2 algorithm.

The CS-TV^2 method can thus facilitate *in situ* structural biology by supporting higher-resolution STA with a limited amount of data and also by improving the visibility of macromolecules in cryo-ET data for target identification. Beyond the results presented here, further studies using CS-TV^2 and alternatives such as wavelet CS-ET methods (Jacob et al., 2021) or other CS methods applied in other imaging techniques, like curvelets (Starck et al., 2002) or shearlets (Kutyniok et al., 2016), should focus on applications on cellular datasets to fully demonstrate the advantages for subnanometer-resolution STA for *in situ* structural biology.

STAR★METHODS

Detailed methods are provided in the online version of this paper and include the following:

- KEY RESOURCES TABLE
- RESOURCE AVAILABILITY
  - Lead contact
  - Materials availability
  - Data and code availability
- EXPERIMENTAL MODEL AND SUBJECT DETAILS
- METHOD DETAILS
  - WBP reconstructions

- ○ CS-TV^2 reconstructions
- ○ Two-dimensional phantom
- ○ CS-TV^2 reconstruction of HBV capsid tomograms
- ○ CS-TV^2 reconstruction of *S. cerevisiae* 80 S ribosomes (EMPIAR-10045)
- ○ CS-TV^2 reconstruction of *C. crescentus* cells
- ○ STA and post-processing for HBV capsids
- ○ STA and post-processing for *S. cerevisiae* 80 S ribosomes (EMPIAR-10045)
- ○ Data visualisation
- ○ Movie creation
- QUANTIFICATION AND STATISTICAL ANALYSIS

SUPPLEMENTAL INFORMATION

Supplemental information can be found online at https://doi.org/10.1016/j.str.2021.12.010.

ACKNOWLEDGMENTS

T.A.M.B. is a recipient of a Sir Henry Dale Fellowship, jointly funded by the Wellcome Trust and the Royal Society (202231/Z/16/Z). T.A.M.B would like to thank the Vallee Research Foundation, the Leverhulme Trust, and the John Fell Fund for support. J.B. is supported by a Medical Research Council graduate studentship (grants MR/K501256/1 and MR/N013468/1). S.M.C. acknowledges support from the Henslow Research Fellowship at Girton College, University of Cambridge, and a University Academic Fellowship at the University of Leeds. Elements of this work were undertaken on ARC3, part of the High Performance Computing facilities at the University of Leeds (United Kingdom). We thank R. Tovey for useful discussions regarding use of CS reconstruction code implementation, Signe Lagercrantz for pre-processing of tomographic data, and Israel S. Fernández for providing a high-resolution cryo-EM density of the *S. cerevisiae* ribosome sample, which was used for creating the EMPIAR-10045 dataset.

AUTHOR CONTRIBUTIONS

Conceptualization, T.A.M.B. and S.M.C.; methodology, J.B., T.A.M.B., and S.M.C.; software, S.M.C; formal analysis, J.B.; investigation, J.B., T.A.M.B., and S.M.C.; resources, T.A.M.B. and S.M.C; writing – original draft, review, and editing, J.B., T.A.M.B., and S.M.C.; visualization, J.B., T.A.M.B., and S.M.C; supervision, T.A.M.B. and S.M.C.; funding acquisition, T.A.M.B. and S.M.C.

DECLARATION OF INTERESTS

The authors declare no competing interests.

Received: March 22, 2021
Revised: October 21, 2021
Accepted: December 22, 2021
Published: January 19, 2022

REFERENCES

Adcock, B., Hansen, A.C., Poon, C., and Roman, B. (2017). Breaking the coherence barrier: a new theory for compressed sensing. In Paper Presented at: Forum of Mathematics, Sigma, *5* (Cambridge University Press), e4.

Aganj, I., Bartesaghi, A., Borgnia, M., Liao, H.Y., Sapiro, G., and Subramaniam, S. (2007). Regularization for inverting the radon transform with wedge consideration. In Paper Presented at: 2007 4th IEEE International Symposium on Biomedical Imaging: From Nano to Macro (IEEE)).

Agulleiro, J., and Fernandez, J.-J. (2011). Fast tomographic reconstruction on multicore computers. Bioinformatics *27*, 582–583.

AlAfeef, A., Bobynko, J., Cockshott, W.P., Craven, A.J., Zuazo, I., Barges, P., and MacLaren, I. (2016). Linear chemically sensitive electron tomography using DualEELS and dictionary-based compressed sensing. Ultramicroscopy *170*, 96–106.

Allegretti, M., Zimmerli, C.E., Rantos, V., Wilfling, F., Ronchi, P., Fung, H.K., Lee, C.-W., Hagen, W., Turoňová, B., Karius, K., et al. (2020). In-cell architecture of the nuclear pore and snapshots of its turnover. Nature *586*, 796–800.

Andersen, A.H., and Kak, A.C. (1984). Simultaneous algebraic reconstruction technique (SART): a superior implementation of the ART algorithm. Ultrason. Imaging *6*, 81–94.

Batenburg, K.J., and Sijbers, J. (2007). DART: a fast heuristic algebraic reconstruction algorithm for discrete tomography. In Paper Presented at: 2007 IEEE International Conference on Image Processing (IEEE), pp. IV–133-IV-136.

Baumeister, W. (2002). Electron tomography: towards visualizing the molecular organization of the cytoplasm. Curr. Opin. Struct. Biol. *12*, 679–684.

Beck, M., and Baumeister, W. (2016). Cryo-electron tomography: can it reveal the molecular sociology of cells in atomic detail? Trends Cell Biol. *26*, 825–837.

Bharat, T.A., and Scheres, S.H. (2016). Resolving macromolecular structures from electron cryo-tomography data using subtomogram averaging in RELION. Nat. Protoc. *11*, 2054–2065.

Bharat, T.A.M., Kureisaite-Ciziene, D., Hardy, G.G., Yu, E.W., Devant, J.M., Hagen, W.J.H., Brun, Y.V., Briggs, J.A.G., and Löwe, J. (2017). Structure of the hexagonal surface layer on Caulobacter crescentus cells. Nat. Microbiol. *2*, 17059.

Bharat, T.A.M., Russo, C.J., Löwe, J., Passmore, L.A., and Scheres, S.H.W. (2015). Advances in single-particle electron cryomicroscopy structure determination applied to sub-tomogram averaging. Structure *23*, 1743–1753.

Bladt, E., Pelt, D.M., Bals, S., and Batenburg, K.J. (2015). Electron tomography based on highly limited data using a neural network reconstruction technique. Ultramicroscopy *158*, 81–88.

Böhning, J., and Bharat, T.A. (2021). Towards high-throughput in situ structural biology using electron cryotomography. Prog. Biophys. Mol. Biol. *160*, 97–103.

Böttcher, B., and Nassal, M. (2018). Structure of mutant hepatitis B core protein capsids with premature secretion phenotype. J. Mol. Biol. *430*, 4941–4954.

Briggs, J.A. (2013). Structural biology in situ–the potential of subtomogram averaging. Curr. Opin. Struct. Biol. *23*, 261–267.

Chen, M., Bell, J.M., Shi, X., Sun, S.Y., Wang, Z., and Ludtke, S.J. (2019). A complete data processing workflow for cryo-ET and subtomogram averaging. Nat. Methods *16*, 1161–1168.

Chen, Y., and Förster, F. (2014). Iterative reconstruction of cryo-electron tomograms using nonuniform fast Fourier transforms. J. Struct. Biol. *185*, 309–316.

Collins, S.M., MacArthur, K.E., Longley, L., Tovey, R., Benning, M., Schönlieb, C.-B., Bennett, T.D., and Midgley, P.A. (2019). Phase diagrams of liquid-phase mixing in multi-component metal-organic framework glasses constructed by quantitative elemental nano-tomography. APL Mater. *7*, 091111.

de la Peña, F., Ostasevicius, T., Fauske, V.T., Burdet, P., Jokubauskas, P., Nord, M., Sarahan, M., Prestat, E., Johnstone, D.N., and Taillon, J. (2017). Electron microscopy (big and small) data analysis with the open source software package HyperSpy. Microsc. Microanalysis *23*, 214–215.

Deng, Y., Chen, Y., Zhang, Y., Wang, S., Zhang, F., and Sun, F. (2016). ICON: 3D reconstruction with 'missing-information'restoration in biological electron tomography. J. Struct. Biol. *195*, 100–112.

Frangakis, A.S., Böhm, J., Förster, F., Nickell, S., Nicastro, D., Typke, D., Hegerl, R., and Baumeister, W. (2002). Identification of macromolecular complexes in cryoelectron tomograms of phantom cells. Proc. Natl. Acad. Sci. U S A. *99*, 14153–14158.

Galaz-Montoya, J.G., Flanagan, J., Schmid, M.F., and Ludtke, S.J. (2015). Single particle tomography in EMAN2. J. Struct. Biol. *190*, 279–290.

Galaz-Montoya, J.G., and Ludtke, S.J. (2017). The advent of structural biology in situ by single particle cryo-electron tomography. Biophys. Rep. *3*, 17–35.

Geng, Z., She, Z., Zhou, Q., Dong, Z., Zhan, F., Zhang, H., Xu, J., Gao, Z., and Dong, Y. (2021). NUDIM: a non-uniform fast Fourier transform based dual-

space constraint iterative reconstruction method in biological electron tomography. J. Struct. Biol. *213*, 107770.

Ghosal, D., Kim, K.W., Zheng, H., Kaplan, M., Truchan, H.K., Lopez, A.E., McIntire, I.E., Vogel, J.P., Cianciotto, N.P., and Jensen, G.J. (2019). In vivo structure of the Legionella type II secretion system by electron cryotomography. Nat. Microbiol. *4*, 2101–2108.

Gilbert, P. (1972). Iterative methods for the three-dimensional reconstruction of an object from projections. J. Theor. Biol. *36*, 105–117.

Goldstein, T., Li, M., Yuan, X., Esser, E., and Baraniuk, R. (2013). Adaptive primal-dual hybrid gradient methods for saddle-point problems. arXiv, 13050546.

Gordon, R., Bender, R., and Herman, G.T. (1970). Algebraic reconstruction techniques (ART) for three-dimensional electron microscopy and X-ray photography. J. Theor. Biol. *29*, 471–481.

Goris, B., Van den Broek, W., Batenburg, K.J., Mezerji, H.H., and Bals, S. (2012). Electron tomography based on a total variation minimization reconstruction technique. Ultramicroscopy *113*, 120–130.

Grünewald, K., Desai, P., Winkler, D.C., Heymann, J.B., Belnap, D.M., Baumeister, W., and Steven, A.C. (2003). Three-dimensional structure of herpes simplex virus from cryo-electron tomography. Science (New York, NY) *302*, 1396–1398.

Guay, M.D., Czaja, W., Aronova, M.A., and Leapman, R.D. (2016). Compressed sensing electron tomography for determining biological structure. Scientific Rep. *6*, 1–14.

Haberfehlner, G., Orthacker, A., Albu, M., Li, J., and Kothleitner, G. (2014). Nanoscale voxel spectroscopy by simultaneous EELS and EDS tomography. Nanoscale *6*, 14563–14569.

Himes, B.A., and Zhang, P. (2018). emClarity: software for high-resolution cryo-electron tomography and subtomogram averaging. Nat. Methods *15*, 955.

Hoffmann, P.C., Bharat, T.A.M., Wozny, M.R., Boulanger, J., Miller, E.A., and Kukulski, W. (2019). Tricalbins contribute to cellular lipid flux and form curved ER-PM contacts that are bridged by rod-shaped structures. Dev. Cell *51*, 488–502 e488.

Huber, R., Haberfehlner, G., Holler, M., Kothleitner, G., and Bredies, K. (2019). Total generalized variation regularization for multi-modal electron tomography. Nanoscale *11*, 5617–5632.

Jacob, M., El Gueddari, L., Lin, J.-M., Navarro, G., Jannaud, A., Mula, G., Bayle-Guillemaud, P., Ciuciu, P., and Saghi, Z. (2021). Gradient-based and wavelet-based compressed sensing approaches for highly undersampled tomographic datasets. Ultramicroscopy *225*, 113289.

Klumpe, S., Fung, H.K., Goetz, S.K., Zagoriy, I., Hampoelz, B., Zhang, X., Erdmann, P.S., Baumbach, J., Mueller, C.W., Beck, M., et al. (2021). A modular platform for streamlining automated cryo-FIB workflows. bioRxiv. https://doi.org/10.1101/2021.05.19.444745.

Kremer, J.R., Mastronarde, D.N., and McIntosh, J.R. (1996). Computer visualization of three-dimensional image data using IMOD. J. Struct. Biol. *116*, 71–76.

Kunz, M., and Frangakis, A.S. (2014). Super-sampling SART with ordered subsets. J. Struct. Biol. *188*, 107–115.

Kutyniok, G., Lim, W.-Q., and Reisenhofer, R. (2016). Shearlab 3D: faithful digital shearlet transforms based on compactly supported shearlets. ACM Trans. Math. Softw. (Toms) *42*, 1–42.

Leary, R., Saghi, Z., Midgley, P.A., and Holland, D.J. (2013). Compressed sensing electron tomography. Ultramicroscopy *131*, 70–91.

Li, L., Han, R., Zhang, Z., Guo, T., Liu, Z., and Zhang, F. (2020). Compressed sensing improved iterative reconstruction-reprojection algorithm for electron tomography. BMC bioinformatics *21*, 1–19.

Li, X., Mooney, P., Zheng, S., Booth, C.R., Braunfeld, M.B., Gubbens, S., Agard, D.A., and Cheng, Y. (2013). Electron counting and beam-induced motion correction enable near-atomic-resolution single-particle cryo-EM. Nat. Methods *10*, 584–590.

Liu, B., Yu, H., Verbridge, S.S., Sun, L., and Wang, G. (2014). Dictionary-learning-based reconstruction method for electron tomography. Scanning: J. Scanning Microscopies *36*, 377–383.

Lučić, V., Förster, F., and Baumeister, W. (2005). Structural studies by electron tomography: from cells to molecules. Annu. Rev. Biochem. *74*, 833–865.

Lučić, V., Rigort, A., and Baumeister, W. (2013). Cryo-electron tomography: the challenge of doing structural biology in situ. J. Cell Biol. *202*, 407–419.

Marko, M., Hsieh, C., Schalek, R., Frank, J., and Mannella, C. (2007). Focused-ion-beam thinning of frozen-hydrated biological specimens for cryo-electron microscopy. Nat. Methods *4*, 215–217.

McMullan, G., Chen, S., Henderson, R., and Faruqi, A. (2009). Detective quantum efficiency of electron area detectors in electron microscopy. Ultramicroscopy *109*, 1126–1143.

Medeiros, J.M., Böck, D., and Pilhofer, M. (2018). Imaging bacteria inside their host by cryo-focused ion beam milling and electron cryotomography. Curr. Opin. Microbiol. *43*, 62–68.

Melia, C.E., and Bharat, T.A. (2018). Locating macromolecules and determining structures inside bacterial cells using electron cryotomography. Biochim. Biophys. Acta (BBA)-Proteins Proteomics *1866*, 973–981.

Pettersen, E.F., Goddard, T.D., Huang, C.C., Meng, E.C., Couch, G.S., Croll, T.I., Morris, J.H., and Ferrin, T.E. (2021). UCSF ChimeraX: structure visualization for researchers, educators, and developers. Protein Sci. *30*, 70–82.

Pfeffer, S., and Mahamid, J. (2018). Unravelling molecular complexity in structural cell biology. Curr. Opin. Struct. Biol. *52*, 111–118.

Pryor, A., Yang, Y., Rana, A., Gallagher-Jones, M., Zhou, J., Lo, Y.H., Melinte, G., Chiu, W., Rodriguez, J.A., and Miao, J. (2017). GENFIRE: a generalized Fourier iterative reconstruction algorithm for high-resolution 3D imaging. Scientific Rep. *7*, 1–12.

Punjani, A., and Fleet, D.J. (2021). 3D variability analysis: resolving continuous flexibility and discrete heterogeneity from single particle cryo-EM. J. Struct. Biol. *213*, 107702.

Punjani, A., Rubinstein, J.L., Fleet, D.J., and Brubaker, M.A. (2017). cryoSPARC: algorithms for rapid unsupervised cryo-EM structure determination. Nat. Methods *14*, 290–296.

Rohou, A., and Grigorieff, N. (2015). CTFFIND4: fast and accurate defocus estimation from electron micrographs. J. Struct. Biol. *192*, 216–221.

Rout, M.P., and Sali, A. (2019). Principles for integrative structural biology studies. Cell *177*, 1384–1403.

Saghi, Z., Divitini, G., Winter, B., Leary, R., Spiecker, E., Ducati, C., and Midgley, P.A. (2016). Compressed sensing electron tomography of needle-shaped biological specimens–Potential for improved reconstruction fidelity with reduced dose. Ultramicroscopy *160*, 230–238.

Saghi, Z., Holland, D.J., Leary, R., Falqui, A., Bertoni, G., Sederman, A.J., Gladden, L.F., and Midgley, P.A. (2011). Three-dimensional morphology of iron oxide nanoparticles with reactive concave surfaces. A compressed sensing-electron tomography (CS-ET) approach. Nano Lett. *11*, 4666–4673.

Sanders, T., Gelb, A., Platte, R.B., Arslan, I., and Landskron, K. (2017). Recovering fine details from under-resolved electron tomography data using higher order total variation ℓ1 regularization. Ultramicroscopy *174*, 97–105.

Scheres, S.H. (2012). RELION: implementation of a Bayesian approach to cryo-EM structure determination. J. Struct. Biol. *180*, 519–530.

Schindelin, J., Arganda-Carreras, I., Frise, E., Kaynig, V., Longair, M., Pietzsch, T., Preibisch, S., Rueden, C., Saalfeld, S., and Schmid, B. (2012). Fiji: an open-source platform for biological-image analysis. Nat. Methods *9*, 676–682.

Schur, F.K., Obr, M., Hagen, W.J., Wan, W., Jakobi, A.J., Kirkpatrick, J.M., Sachse, C., Kräusslich, H.G., and Briggs, J.A. (2016). An atomic model of HIV-1 capsid-SP1 reveals structures regulating assembly and maturation. Science (New York, NY) *353*, 506–508.

Scott, M., Chen, C.-C., Mecklenburg, M., Zhu, C., Xu, R., Ercius, P., Dahmen, U., Regan, B., and Miao, J. (2012). Electron tomography at 2.4-ångström resolution. Nature *483*, 444–447.

Shi, X., Chen, M., Yu, Z., Bell, J.M., Wang, H., Forrester, I., Villarreal, H., Jakana, J., Du, D., Luisi, B.F., et al. (2019). In situ structure and assembly of the multidrug efflux pump AcrAB-TolC. Nat. Commun. *10*, 1–6.

CellPress
OPEN ACCESS

Song, K., Comolli, L.R., and Horowitz, M. (2012). Removing high contrast artifacts via digital inpainting in cryo-electron tomography: an application of compressed sensing. J. Struct. Biol. *178*, 108–120.

Sorzano, C.O.S., Vargas, J., Otón, J., de la Rosa-Trevín, J., Vilas, J., Kazemi, M., Melero, R., Del Caño, L., Cuenca, J., and Conesa, P. (2017). A survey of the use of iterative reconstruction algorithms in electron microscopy. Biomed. Research International *2017*, 6482567.

Starck, J.-L., Candès, E.J., and Donoho, D.L. (2002). The curvelet transform for image denoising. IEEE Trans. image Process. *11*, 670–684.

Sulkowski, N.I., Hardy, G.G., Brun, Y.V., and Bharat, T.A. (2019). A multiprotein complex anchors adhesive holdfast at the outer membrane of Caulobacter crescentus. J. Bacteriol. *201*, e00112–e00119.

Tang, G., Peng, L., Baldwin, P.R., Mann, D.S., Jiang, W., Rees, I., and Ludtke, S.J. (2007). EMAN2: an extensible image processing suite for electron microscopy. J. Struct. Biol. *157*, 38–46.

Tegunov, D., and Cramer, P. (2019). Real-time cryo-electron microscopy data preprocessing with Warp. Nat. Methods *16*, 1146–1152.

Tegunov, D., Xue, L., Dienemann, C., Cramer, P., and Mahamid, J. (2021). Multi-particle cryo-EM refinement with M visualizes ribosome-antibiotic complex at 3.5 Å in cells. Nat. Methods *18*, 186–193.

Tovey, R., Johnstone, D.N., Collins, S.M., Lionheart, W.R., Midgley, P.A., Benning, M., and Schönlieb, C.-B. (2020). Scanning electron diffraction tomography of strain. Inverse Probl. *37*, 015003.

Turoňová, B., Marsalek, L., Davidovič, T., and Slusallek, P. (2015). Progressive stochastic reconstruction technique (PSRT) for cryo electron tomography. J. Struct. Biol. *189*, 195–206.

Turoňová, B., Schur, F.K., Wan, W., and Briggs, J.A. (2017). Efficient 3D-CTF correction for cryo-electron tomography using NovaCTF improves subtomogram averaging resolution to 3.4 angstrom. J. Struct. Biol. *199*, 187–195.

Van Aarle, W., Palenstijn, W.J., De Beenhouwer, J., Altantzis, T., Bals, S., Batenburg, K.J., and Sijbers, J. (2015). The ASTRA Toolbox: a platform for advanced algorithm development in electron tomography. Ultramicroscopy *157*, 35–47.

Van der Walt, S., Schönberger, J.L., Nunez-Iglesias, J., Boulogne, F., Warner, J.D., Yager, N., Gouillart, E., and Yu, T. (2014). scikit-image: image processing in Python. PeerJ *2*, e453.

Villa, E., Schaffer, M., Plitzko, J.M., and Baumeister, W. (2013). Opening windows into the cell: focused-ion-beam milling for cryo-electron tomography. Curr. Opin. Struct. Biol. *23*, 771–777.

von Kugelgen, A., Tang, H., Hardy, G.G., Kureisaite-Ciziene, D., Brun, Y.V., Stansfeld, P.J., Robinson, C.V., and Bharat, T.A.M. (2020). In situ structure of an intact lipopolysaccharide-bound bacterial surface layer. Cell *180*, 348–358 e315.

Wan, W., and Briggs, J. (2016). Cryo-electron tomography and subtomogram averaging. In Methods in Enzymology (Elsevier), pp. 329–367.

Wang, Q., Mercogliano, C.P., and Löwe, J. (2011). A ferritin-based label for cellular electron cryotomography. Structure *19*, 147–154.

Watanabe, R., Buschauer, R., Böhning, J., Audagnotto, M., Lasker, K., Lu, T.-W., Boassa, D., Taylor, S., and Villa, E. (2020). The in situ structure of Parkinson's disease-linked LRRK2. Cell *182*, 1508–1518.e1516.

Weiss, G.L., Kieninger, A.-K., Maldener, I., Forchhammer, K., and Pilhofer, M. (2019). Structure and function of a bacterial gap junction analog. Cell *178*, 374–384.e315.

Wu, X., Zeng, X., Zhu, Z., Gao, X., and Xu, M. (2019). Template-based and template-free approaches in cellular cryo-electron tomography structural pattern mining. In Computational Biology (Codon Publications), pp. 175–186.

Yan, R., Venkatakrishnan, S.V., Liu, J., Bouman, C.A., and Jiang, W. (2019). MBIR: a cryo-ET 3D reconstruction method that effectively minimizes missing wedge artifacts and restores missing information. J. Struct. Biol. *206*, 183–192.

Yang, Y., Chen, C.-C., Scott, M., Ophus, C., Xu, R., Pryor, A., Wu, L., Sun, F., Theis, W., and Zhou, J. (2017). Deciphering chemical order/disorder and material properties at the single-atom level. Nature *542*, 75–79.

Zachs, T., Schertel, A., Medeiros, J., Weiss, G.L., Hugener, J., Matos, J., and Pilhofer, M. (2020). Fully automated, sequential focused ion beam milling for cryo-electron tomography. Elife *9*, e52286.

Zhong, E.D., Bepler, T., Berger, B., and Davis, J.H. (2021). CryoDRGN: reconstruction of heterogeneous cryo-EM structures using neural networks. Nat. Methods *18*, 176–185.

STAR★METHODS

KEY RESOURCES TABLE

REAGENT or RESOURCE	SOURCE	IDENTIFIER
Deposited data		
Caulobacter crescentus stalk CS tomographic reconstruction	This study	EMD-13881
Hepatitis B capsid WT	(Böttcher and Nassal, 2018)	PDB-ID 6HTX
S. cerevisiae 80S ribosome dataset	(Bharat and Scheres, 2016)	EMPIAR-10045
Software and algorithms		
CS-TV^2 reconstruction algorithm	(Tovey et al., 2020)	https://github.com/robtovey/ToveyTomoTools
ASTRA Toolbox	(Van Aarle et al., 2015)	https://www.astra-toolbox.com/
SciKit-Image (Version 0.16.2)	(Van der Walt et al., 2014)	https://scikit-image.org/
HyperSpy (Version 1.5.2)	(de la Peña et al., 2017)	https://hyperspy.org/
ChimeraX (Version 1.0.0)	(Pettersen et al., 2021)	https://www.cgl.ucsf.edu/chimerax/
IMOD (Version 4.9.10)	(Kremer et al., 1996)	https://bio3d.colorado.edu/imod/
ImageJ (Version 2.1.0/1.53c)	(Schindelin et al., 2012)	https://imagej.net/software/fiji/
RELION (Version 3.1)	(Bharat et al., 2015; Scheres, 2012)	https://github.com/3dem/relion
MATLAB (Version 2018b)	MathWorks	https://mathworks.com/products/matlab.html
CTFFIND 4	(Rohou and Grigorieff, 2015)	https://grigoriefflab.umassmed.edu/ctffind4

RESOURCE AVAILABILITY

Lead contact

Further information and requests for resources and reagents should be directed to and will be fulfilled by the Lead Contact, Tanmay Bharat (tanmay.bharat@path.ox.ac.uk).

Materials availability

This study did not generate new unique materials.

Data and code availability

A cropped CS-TV^2 tomogram of the S-layer as shown in Figure 4 has been deposited at the Electron Microscopy Databank (EMDB) with the accession code EMD-13881. This paper also uses existing, publicly available data, whose accession numbers are listed in the Key Resources Table. This paper does not report original code. Any additional information required to reanalyse the data reported in this paper is available from the lead contact upon reasonable request.

EXPERIMENTAL MODEL AND SUBJECT DETAILS

All data are generated from the datasets provided in the Key Resources Table.

METHOD DETAILS

WBP reconstructions

All WBP reconstructions were performed in IMOD. Prior to reconstruction of tomograms for STA, defoci of tilt images were estimated using CTFFIND4 (Rohou and Grigorieff, 2015), and the CTF compensated via phase-flipping as implemented in IMOD. WBP subtomograms were cropped out of unbinned WBP tomograms (pixel size 2.17 Å) using RELION (Scheres, 2012).

CS-TV^2 reconstructions

All CS reconstructions were carried out using second order total-variation (TV^2) regularization with a primal-dual hybrid gradient algorithm (Goldstein et al., 2013), implemented in Python using the ASTRA Toolbox for a projector (Van Aarle et al., 2015), enforcing non-negativity in the reconstructions. In contrast to other CS implementations applied to cryotomography that make use of image

sparsity in two-dimensional slice-by-slice reconstructions in the Fourier transform domain (Deng et al., 2016), this implementation uses real-space projection and fully three-dimensional CS-TV^2 calculations. Real space projection operators avoid interpolation required in Fourier space algorithms (Goris et al., 2012), and 3D TV implementations have been shown to improve reconstruction quality (Haberfehlner et al., 2014) as they maintain a consistent relative weighting of the transform term for the entire reconstruction volume and also reinforce the 3D connected structure. The implementation of the component parts has been reported previously (Tovey et al., 2020) and the code is available via Github (https://github.com/robtovey/ToveyTomoTools). Briefly, the code for CS-TV^2 reconstructions consists of a Python framework, coded in NumPy and SciPy to interface with the ASTRA toolbox and to implement linear algebra operations required for the primal-dual hybrid gradient algorithm (Goldstein et al., 2013). The primal-dual hybrid gradient algorithm, briefly, recasts the CS-ET algorithm, which seeks to minimize both the error of the re-projected tomographic volume with respect to the tilt-series data as well as the second order total variation, to a saddle-point problem seeking to minimize the data fidelity term and setting the total variation term derived from finite differences instead as a mathematically equivalent maximization over a 'dual' variable. The algorithm then takes iterative gradient-guided steps to optimize the solution to the overall CS-ET reconstruction problem with established guarantees on convergence.

Two-dimensional phantom

The two-dimensional phantom was derived from a density map of a molecular model of the HBV capsid. For the phantom, a full 180° angular range was used with a tilt increment of 1°. Calculations were carried out in open-source Python packages: Phantom calculations used the forward and inverse Radon transform in Scikit-Image (Van der Walt et al., 2014). Poisson noise was added to emulate experimental conditions with low signal-to-noise ratio at low electron fluence using HyperSpy (de la Peña et al., 2017). Displayed gradients were calculated as finite differences using NumPy.

CS-TV^2 reconstruction of HBV capsid tomograms

CS-TV^2 promotes reconstructions using the entire three-dimensional TV^2-sparsity. In recognition of the increased memory requirements, advantages of parallelization, and the independence of each HBV capsid volume, CS reconstructions of HBV capsids were carried out on a particle-by-particle basis, that is, each capsid was reconstructed separately. Tilt series were taken from the HBV capsid dataset described in (Bharat et al., 2015). Manually clicked particle coordinates (used above in WBP) were used to center each particle on the tilt-axis using geometrically determined lateral shifts with subpixel precision using the HyperSpy Python package, resulting in a 300 × 300 pixel^2 area for each HBV capsid. Reconstructions were performed with a box size of 300×300×300 voxels (pixel size 2.17 Å). No down-sampling was applied to this or any of the other datasets. In order to use the CS-TV^2 algorithm with a non-negative projector and to reinforce the compact support of the particles, the intensities were inverted: first, an area containing no particles in any image in the tilt-series was selected and used to calculate the background intensity value at each tilt. The intensities were inverted relative to this value and scaled to give an intensity maximum of one. The number of iterations to achieve acceptable convergence was examined; reconstructions at 2000 iterations did not show significant improvements over reconstructions at 200 iterations. First order TV reconstructions were examined but exhibited either convergence to zero throughout the reconstruction volume or highly blurred features. Reconstructions of 300 × 300×300 voxel tomograms at 200 iterations took ~20 min each on a local workstation with a single GPU. Due to the inherent independence of each reconstruction, the reconstructions were also fully parallelized on a GPU-integrated cluster on the ARC3 cluster at the University of Leeds, where a typical reconstruction at 200 iterations using P100 GPU nodes took <35 min. Further optimisation of the algorithm implementation as well as increasing computing power may reduce these times further. To remove artifacts resulting from the CS-TV^2 reconstruction near the edge of the subtomograms, boxes of 216×216×216 pixels were cropped out for STA refinements. All particles were normalized within RELION before subtomogram averaging.

CS-TV^2 reconstruction of *S. cerevisiae* 80 S ribosomes (EMPIAR-10045)

CS reconstructions of *S. cerevisiae* 80 S ribosomes (EMPIAR-10045) were carried out using a similar approach to the HBV capsids. Manually clicked particle coordinates (used above in WBP) were used to center each particle on the tilt-axis using geometrically determined lateral shifts with subpixel precision using the HyperSpy Python package, resulting in a 280×280 pixel^2 area for each ribosome. Reconstructions were performed with a box size of 280×280×280 voxels (pixel size 2.17 Å). Due to the higher density of ribosomes, areas free of particles at all tilts were not identified for background subtraction. Instead, areas at 0° tilt near the centre of the hole in the support film were identified containing no ribosomes. Then, assuming a film of ice slab of constant thickness, the tilt-dependent background intensities were calculated geometrically and subtracted from each tilt separately. After inversion, areas of thickness greater than this ice region near the center of the hole in the support film were positive and compatible with the non-negative projector for CS reconstructions.

CS-TV^2 reconstruction of *C. crescentus* cells

Tilt series data of *C. crescentus* cells from a previous study (Bharat et al., 2017) were used for tomographic reconstruction. Due to the memory requirements for three-dimensional CS-TV^2 reconstructions, a 'chunk'-by-'chunk' approach was used for *C. crescentus* stalk reconstructions, mimicking a conventional slice-by-slice approach of a series of two-dimensional reconstructions but incorporating CS-TV^2 constraints in the third dimension of the reconstruction volume. Intensities, as with HBV capsid reconstructions, were inverted with reference to an area in the tilt-series not containing any of the *C. crescentus* stalk. Chunks were 600 or 800 pixels across

and 100 pixels in the direction parallel the tilt axis, giving reconstruction volumes of 600×600×100 or 800×800×100 voxels. The larger size was used where the physical dimensions of the stalk in projection required the increased latter dimensions. Geometric shifts were applied to center each chunk on the tilt-axis as for the HBV particle-by-particle reconstructions. Because the entire object volume was not contained within a single reconstruction volume, chunks were selected to overlap by 25 pixels with each of the adjacent chunks. Any errors arising from the CS-TV^2 calculations at the boundary were therefore excluded from the final reconstruction volume (a total of 50 pixels excluded from each reconstruction volume, with the exception of the first and last 25 pixels, which were retained as these had no overlap with a further chunk). Each reconstruction chunk was then stitched with overlap regions removed, with geometric shifts inverted to return the chunks to the original relative locations with subpixel precision, using shift functions in the HyperSpy Python package. For comparison of computing time with sub-volume approach, execution of this chunk-by-chunk approach on the ARC3 cluster at the University of Leeds typically took <45 minutes for 200 iterations using P100 GPU nodes. Reconstructions were carried out with the weighting parameter λ=0.005, refined by visual inspection of a representative reconstruction for a single chunk at varying parameter values.

For the *C. crescentus* cell presented in Figure S4, the background subtraction step followed the same procedure as for the stalk. A similar chunk-by-chunk approach was used, with a chunk size of 1770×3058×16 voxels, with 16 pixels in the direction along the tilt axis. This chunk size was selected to balance RAM requirements with the larger volume. The weighting parameter was likewise adjusted to λ=0.0015, determined from visual inspection of a single chunk reconstruction for varying parameter values as for the stalk reconstructions.

STA and post-processing for HBV capsids

Previous work on the HBV capsid dataset (Bharat et al., 2015) in our laboratory showed that three tomograms of the dataset were sufficient to resolve α-helices in the capsid protein. Thus, we performed all subtomogram averaging (STA) work with the 188 particles manually picked using IMOD (Kremer et al., 1996) from those three tomograms. Subsets of particles were randomly chosen using the GNU *shuf* utility on the particle lines of the respective RELION star files. Subsets were created sequentially from larger datasets, i.e. the 141 particle dataset was obtained from the 188 particle dataset, the 94 particle dataset was created from the previously obtained 141 particle dataset, and so on.

All STA was performed in RELION (Bharat and Scheres, 2016; Scheres, 2012). Manual star files were created for CS particles to enable implementation into the RELION pipeline. Since CS requires CTF-corrected tilt series, per-particle CTF models in RELION were not used, and replaced by a model considering just the missing wedge. Parameters of the refinement in the "Refine3D" program were unchanged between refinements of CS-TV^2 and WBP subtomograms. An atomic model of the HBV capsid (PDB 6HTX) was turned into an EM density using the Chimera molmap command and a heavily lowpass filtered map was used as a reference for all refinements in the case of HBV capsids. The same map (unfiltered) was used as a basis for model-vs-map FSC estimations. RELION was used for all FSC calculations. The mask used for refinement was a hollow spherical mask encompassing the HBV capsid with an added soft edge. Post-processing was performed using the alignment mask, which was consistent between all runs. Maps showing secondary structure were lowpass-filtered to their respective estimated resolutions as determined via gold-standard FSC (cut-off 0.143, independent half-maps). STA maps not showing any secondary structure were lowpass-filtered to 20 Å to allow better comparability of map quality – this is because an EM map of the HBV capsid lowpass-filtered to 20 Å is difficult to visually distinguish from a map lowpass-filtered to 30 Å, and to indicate residual noise between 20-30 Å in the WBP map as shown in Figure 3. Suitable B-factors were determined manually for both WBP and CS maps for HBV maps. We found automatic B-factor determination based on the Guinier plot to be not applicable for B-factor sharpening of the CS reconstructions. B-factors were chosen so they resulted in high visibility of molecular features without appearance of significant high-frequency noise within the mask. Generally, CS reconstructions required considerably larger B-factors, due to the relative down-weighing of high-frequency information in the raw average. The application of higher B-factors for CS reconstructions was possible due to the low amounts of high-frequency noise in the map, compared to WBP. The spherical alignment mask was applied for all FSC calculations. The data was not down-sampled during any step. For model-vs-map FSC calculations of a single HBV capsid particle against the near-atomic EM density, orientations and shifts that centre and orient the capsid particle towards the reference, obtained through STA of the whole dataset, were applied.

STA and post-processing for *S. cerevisiae* 80 S ribosomes (EMPIAR-10045)

Data was generally processed as mentioned above for HBV capsid data, with the following differences in the workflow. A soft mask based on a ribosome density was used for alignment and was consistent between CS-TV^2 and WBP subtomogram averaging. Maps in Figure S3 were lowpass-filtered to 15 Å for both CS-TV^2 and WBP, according to gold-standard resolution. For FSC calculations, a 3.7 Å resolution SPA reconstruction of the same sample (Bharat and Scheres, 2016) was employed. Masks for FSC calculations were consistent between WBP and CS-TV^2. RELION was used for all FSC calculations.

Data visualisation

FRC profiles for phantom calculations were carried out in ImageJ using the BIOP plugin. FSC and particle number versus resolution plots were created in MATLAB R2018b (MathWorks). Radial averaging was performed and plotted in MATLAB. EM maps were visualized in ChimeraX (Pettersen et al., 2021) or IMOD as indicated. Atomic models were rigid body-fitted in ChimeraX for visualisation purposes. Tomograms were visualized in IMOD using auto-contrast, except for the *C. crescentus* stalk CS-TV^2 tomogram, where contrast was adjusted manually to increase feature visibility. Generally, CS reconstructions of cellular data required manual

adjustment of contrast for ideal visualisation. For comparison with CS tomograms, WBP tomograms were filtered where indicated with a 3-sigma Gaussian 2D filter and a lowpass-filter in ImageJ ('FFT-filter') with a 10 pixel radius, which was considered ideal for visualisation. Data were not binned for visualisation at any step.

Movie creation

Movies were created in ChimeraX and Fiji (Schindelin et al., 2012) from images of tomographic slices written out in IMOD. The contrast in the *C. crescentus* tomogram was changed from the image in Figure 4C to allow better visualisation of features throughout the movie.

QUANTIFICATION AND STATISTICAL ANALYSIS

The methods of statistical analysis are provided in the Method Details and Supporting Information.

Structure, Volume *30*

Supplemental Information

Compressed sensing for electron cryotomography and high-resolution subtomogram averaging of biological specimens

Jan Böhning, Tanmay A.M. Bharat, and Sean M. Collins

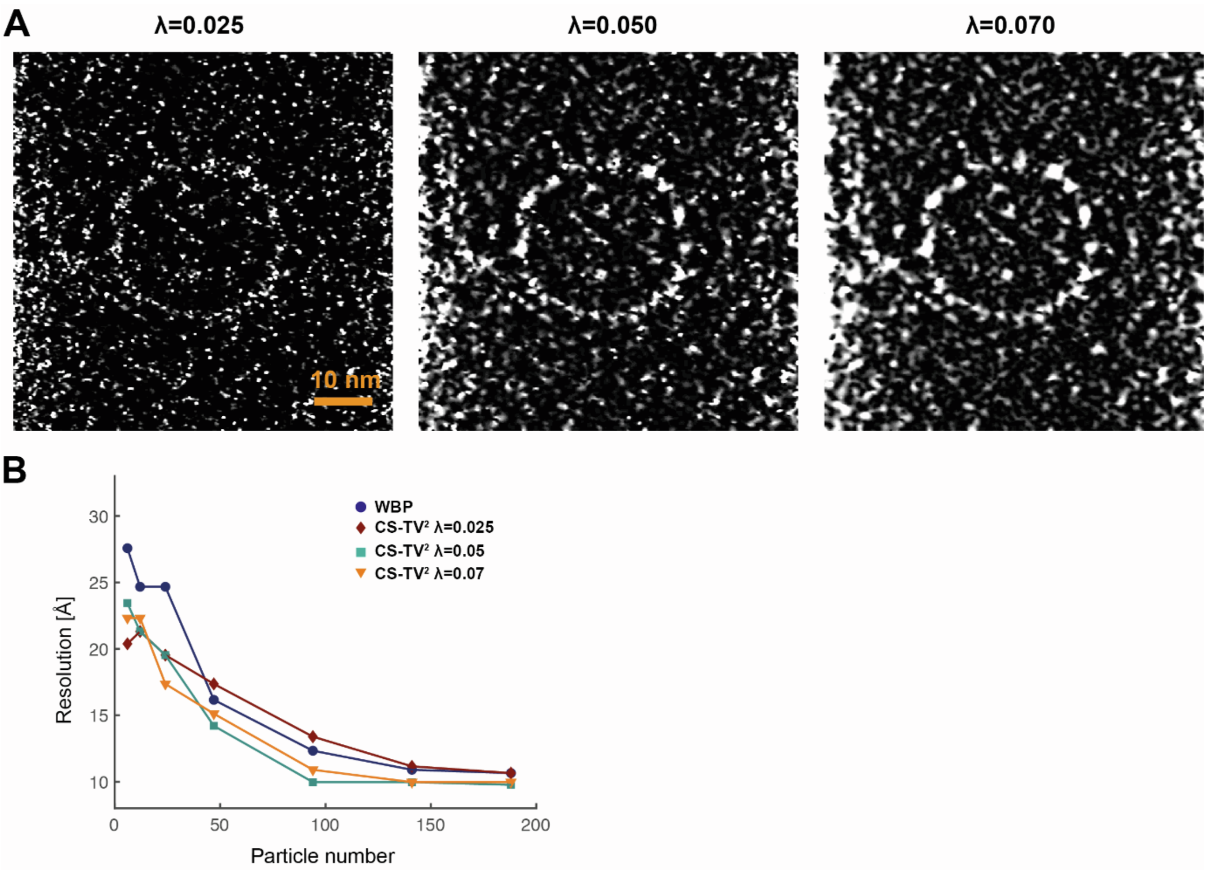

Figure S1. Testing regularization (λ) parameters for CS-TV^2 reconstruction, related to Figure 2.

A) Tomographic slices of reconstructed CS-TV^2 subtomograms with three different regularization parameters. λ=0.05 was selected for the majority of this study. B) Particle number-vs-resolution plot according to the gold-standard criterion indicating self-consistency between half-maps, with datasets reconstructed with different λ parameters. λ=0.05 was chosen as it showed the highest self-consistency between half maps for the full dataset. For resolutions higher than 11 Å, α-helical features could be clearly identified in the corresponding EM density. We speculate that different λ parameters may be suitable for different data amounts, i.e. more regularization might be useful for smaller data amounts and vice versa. While gold-standard FSC was used to find the ideal λ value, model-vs-map FSC were used to validate the results shown in Figure 2.

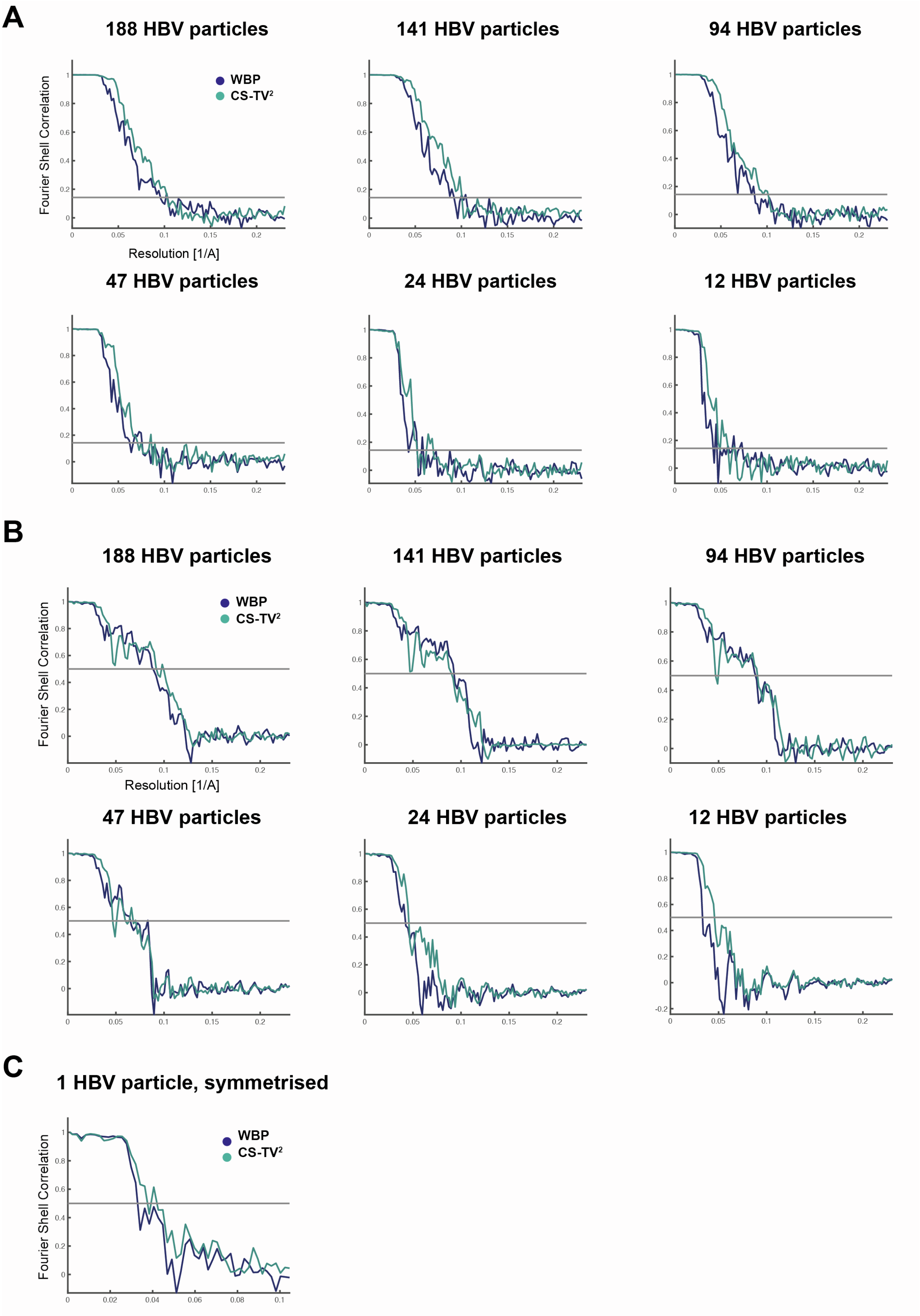

Figure S2. Gold-standard and model-vs-map FSC plots, related to Figure 3.

A) Gold-standard FSC plots for STA maps obtained from averaging of varying numbers of HBV capsid particles. The resolution criterion (FSC=0.143) is indicated. B) Model-vs-map FSC plots versus a density map created from an atomic model (PDB 6HTX). Horizontal line at 0.5 FSC is drawn. B) Model-vs-map FSC plot as in A) for a single subtomogram reconstructed with either WBP or CS-TV^2.

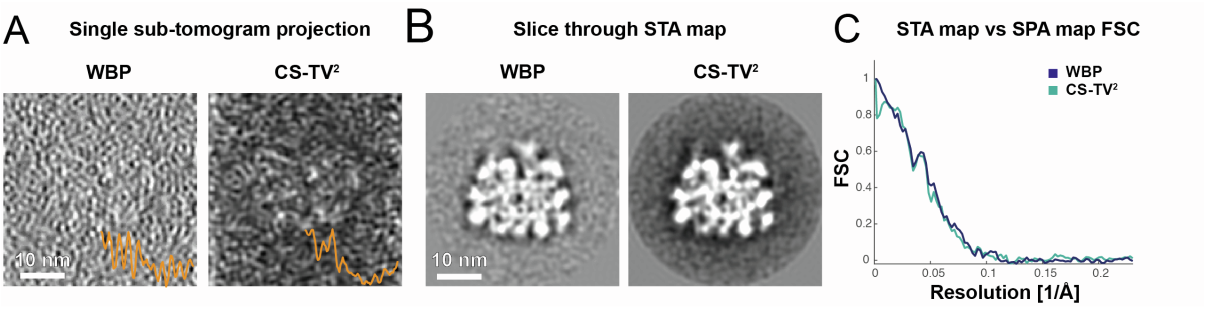

Figure S3. STA of an *S. cerevisiae* ribosome dataset (EMPIAR 10045) reconstructed with CS-TV² and WBP (control), related to Figure 3.

A) Projection image of a single ribosome subtomogram. CS-TV² allows clearer visualisation of the ribosome against the background. Radial averaging (orange line) shows significant variation in intensity for the WBP reconstructions, whereas increased intensity near the centre of the particle is visible for the CS-TV² reconstruction. B) STA map from all particles in the dataset, excluding those too close to the edge for CS-TV² reconstructions. C) FSC against a 3.7 Å cryo-EM map obtained through single particle analysis of the same preparation of specimen, indicating comparable performance between WBP and CS-TV². The same λ parameter (λ=0.05) as the HBV dataset was used for this dataset.

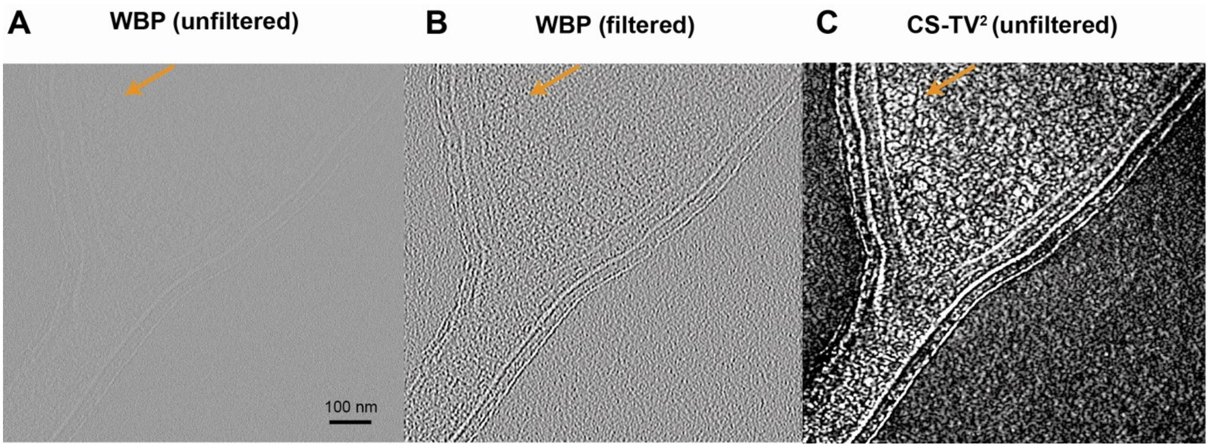

Figure S4. Cryo-ET of a *C. crescentus* cell, related to Figure 4.

A) Unfiltered WBP reconstruction, B) 3-sigma Gaussian and 45-Å lowpass-filtered WBP reconstruction, and C) unfiltered CS-TV^2-reconstruction (λ=0.0015) of the cell body of a *C. crecentus* cell. A putative polysome is indicated by an arrow. CS-TV^2 allows clear visualisation of individual ribosomes without any additional filter or loss of high-frequency information (shown in Figures 2-3).
